# Neuronal methylome reveals CREB-associated neuro-axonal impairment in multiple sclerosis

**DOI:** 10.1186/s13148-019-0678-1

**Published:** 2019-05-30

**Authors:** Lara Kular, Maria Needhamsen, Milena Z. Adzemovic, Tatiana Kramarova, David Gomez-Cabrero, Ewoud Ewing, Eliane Piket, Jesper Tegnér, Stephan Beck, Fredrik Piehl, Lou Brundin, Maja Jagodic

**Affiliations:** 1 Department of Clinical Neuroscience, Center for Molecular Medicine, Karolinska Institutet, Stockholm, Sweden; 2 Department of Medicine, Unit of Computational Medicine, Center for Molecular Medicine, Karolinska Institutet, Solna, Sweden; 3 Mucosal and Salivary Biology Division, King’s College London Dental Institute, London, SE1 9RT UK; 4 Translational Bioinformatics Unit, Navarrabiomed, Complejo Hospitalario de Navarra (CHN), Universidad Pública de Navarra (UPNA), IdiSNA, Pamplona, Spain; 5 Biological and Environmental Sciences and Engineering Division, Computer, Electrical and Mathematical Sciences and Engineering Division, King Abdullah University of Science and Technology, Thuwal, 23955 Saudi Arabia; 6 Medical Genomics, UCL Cancer Institute, University College London, London, UK; 7 Department of Neurology, Karolinska University Hospital, Stockholm, Sweden

**Keywords:** Multiple sclerosis, Neurons, DNA methylation, DNA hydroxymethylation, Axonal guidance, Synaptic plasticity, CREB, Neurodegeneration

## Abstract

**Background:**

Due to limited access to brain tissue, the precise mechanisms underlying neuro-axonal dysfunction in neurological disorders such as multiple sclerosis (MS) are largely unknown. In that context, profiling DNA methylation, which is a stable and cell type-specific regulatory epigenetic mark of genome activity, offers a unique opportunity to characterize the molecular mechanisms underpinning brain pathology in situ. We examined DNA methylation patterns of neuronal nuclei isolated from post-mortem brain tissue to infer processes that occur in neurons of MS patients.

**Results:**

We isolated subcortical neuronal nuclei from post-mortem white matter tissue of MS patients and non-neurological controls using flow cytometry. We examined bulk DNA methylation changes (total *n* = 29) and further disentangled true DNA methylation (5mC) from neuron-specific DNA hydroxymethylation (5hmC) (*n* = 17), using Illumina Infinium 450K arrays. We performed neuronal sub-type deconvolution using glutamate and GABA methylation profiles to further reduce neuronal sample heterogeneity. In total, we identified 2811 and 1534 significant (genome-wide adjusted *P* value < 0.05) differentially methylated and hydroxymethylated positions between MS patients and controls. We found striking hypo-5mC and hyper-5hmC changes occurring mainly within gene bodies, which correlated with reduced transcriptional activity, assessed using published RNAseq data from bulk brain tissue of MS patients and controls. Pathway analyses of the two cohorts implicated dysregulation of genes involved in axonal guidance and synaptic plasticity, with meta-analysis confirming CREB signalling as the most highly enriched pathway underlying these processes. We functionally investigated DNA methylation changes of CREB signalling-related genes by immunohistofluoresence of phosphorylated CREB in neurons from brain sections of a subcohort of MS patients and controls (*n* = 15). Notably, DNA methylation changes associated with a reduction of CREB activity in white matter neurons of MS patients compared to controls.

**Conclusions:**

Our data demonstrate that investigating 5mC and 5hmC modifications separately allows the discovery of a substantial fraction of changes occurring in neurons, which can escape traditional bisulfite-based DNA methylation analysis. Collectively, our findings indicate that neurons of MS patients acquire sustained hypo-5mC and hyper-5hmC, which may impair CREB-mediated neuro-axonal integrity, in turn relating to clinical symptoms.

**Electronic supplementary material:**

The online version of this article (10.1186/s13148-019-0678-1) contains supplementary material, which is available to authorized users.

## Background

Multiple sclerosis (MS), a leading cause of neurological disability in young adults, is a chronic inflammatory and neurodegenerative disease of the central nervous system (CNS) characterized by autoimmune destruction of myelin and subsequent neuronal death [1]. Although the cause of MS remains largely unknown, accumulating data support the notion of MS being a complex disease influenced by genetic [2] (primarily affecting immune genes) and environmental factors [3]. While major advances have been made in understanding immune dysfunction in early phases of MS, leading to increasingly more effective disease modulatory treatments, the pathological basis of late-stage brain pathology is still largely unknown. The disease pathology is characterized by episodic inflammatory demyelination and axonal injury, which translates into clinical symptoms such as sensory, motor, visual and coordination deficits, as well as complex cognitive disturbances [4]. Apart from focal lesions, neuroimaging [5] and histopathological investigations have revealed association between seemingly unaffected normal-appearing white matter (NAWM) integrity and cognitive impairment [6–8]. Evidence suggests that the degree of axonal degeneration rather than demyelination in corticospinal tracts is the major determinant of clinical motor disability [9]. Neuroradiological measures, such as global and regional brain atrophy, are also correlated to disability status, underscoring the neurodegenerative aspect of MS [10, 11]. Altogether, the MS paradigm proposes that exhaustion of neuro-axonal reserve capacity and compensatory mechanisms caused by energy failure, glutamate excitotoxicity and ionic imbalance, among others, will ultimately result in permanent neurological sequelae [12]. However, due to limited accessibility of the CNS, mechanisms underpinning neuronal dysfunction in MS patients remain largely unresolved, further hindering clinical translation for the care of progressive patients.

One way to tackle this challenge is to exploit the remarkable properties of epigenetic modifications, such as DNA methylation, which lies at the interface between internal (genes) and external (microenvironment) cues. DNA methylation is a stable epigenetic mark that can impact gene regulation and/or reflect genome activity and can be accurately quantified at a single-nucleotide resolution in a genome-wide manner post-mortem. As such, DNA methylation offers the unique possibility to interrogate the genome activity underlying cellular dysfunction in conventionally inaccessible organs from autopsy tissue. DNA methylation is acquired and maintained by the action of DNA methyltransferases (DNMTs) that catalyse the covalent addition of a methyl group to cytosine (5mC). Inversely, active demethylation is catalysed by the Ten-eleven translocation (TET) family of enzymes. Importantly, the brain methylome is highly complex due to tissue- and cell type-specific variation between grey matter (GM) and white matter (WM), functionally distinct regions of the brain [13, 14] and cell types [15]. Moreover, the brain and more specifically neurons are enriched in regulatory non-canonical DNA modifications such as CpG hydroxymethylation (5hmC) [15]. A growing body of evidence suggests that in addition to its crucial role in brain development and function [15], DNA methylation could be implicated in MS brain pathology as well [16]. Nevertheless, the majority of studies of DNA methylation in healthy and diseased brain have been performed on mixed cell populations (bulk tissue) or on total DNA methylation modifications (bulk DNA methylation reflecting 5mC + 5hmC), potentially hindering the capture of complex epigenome architecture in neurons in situ.

Despite clinico-pathological evidence of accumulating neuro-axonal impairment occurring over the MS disease course, the precise mechanisms underpinning neuronal dysfunction in MS are still largely unknown. In the present study, we explored the potential of DNA methylation as a readout of neuronal genome activity in post-mortem brain tissue of MS patients and non-neurological controls. Using a strategy combining isolation of neuronal nuclei and array-based 5mC and 5hmC technology, we here present an analysis of neuronal methylome in MS patients, followed by comparison of observed changes with functional outcomes.

## Results

### Investigation of bulk DNA methylation changes in neurons from MS patients

We first evaluated potential sources of heterogeneity in brain tissue that could impact DNA methylation variability. To that end, we conducted genome-wide DNA methylation analyses using Illumina Infinium 450K Human Methylation Beadchip arrays (450K) on bisulfite (BS)-treated genomic DNA in NeuN^+^-sorted neuronal nuclei (> 99% purity) as well as the corresponding unsorted bulk tissue from the same fresh-frozen post-mortem tissue blocks (Additional file 1: Figure S1). The analysed tissue comprised various lesion phenotypes as well as NAWM originating from the same individual (Table 1, Additional file 2). We tested the effect of lesion phenotype, degree of myelination (GM/WM) and cell heterogeneity on DNA methylation values, estimated as *β*-values for each CpG site. As expected, tissue composition (neurons vs. bulk) had the largest influence on DNA methylation profiles (Additional file 1: Figure S1). Analyses further suggested DNA methylation differences between lesion phenotypes (Additional file 1: Figure S1). To account for neuronal subtypes, we conducted cell type deconvolution stratifying for glutamatergic (GLU) and GABAergic neurons based on previously reported differentially methylated CpG sites between NeuN^+^/SOX6^−^ (GLU) and NeuN^+^/SOX6^+^ (GABA) neuronal nuclei [17] and found that neuronal subtype proportion indeed had an impact on DNA methylation profiles (see the “Methods” section and Additional file 1: Figure S2).

**Table 1:** Description of pilot samples and study cohorts

	Pilot samples		Cohort 1		Cohort 2		Cohort IF	
Group	Case	Control	Case	Control	Case	Control	Case	Control
*N* individuals^a^	1	1	5	5	10	7	7	8
Sex ratio (F/M)	1:0	0:1	4:1	1:4	7:3	3:4	6:1	3:5
Age (mean ± SD)	42	92	45.4 ± 4.5	68.4 ± 21.6	60.3 ± 12.4	78.5 ± 7.9	63.6 ± 12.8	78.2 ± 5.1
PMI (mean ± SD)	11	13	17 ± 8.7	21 ± 7.4	17 ± 9.3	11 ± 11.4	15 ± 8.0	23 ± 3.7
*N* brain samples	4	1	7	5	10	7	7	8
Type lesion (*N*)	AL (1)		AL (2)		–		–	
	CAL (1)		–		–		–	
	CL (1)		CL (2)		CL (2)		–	
	NAWM (1)		NAWM (3)		NAWM (8)		NAWM (7)	
Sample type	Bulk tissue and neurons		Neurons		Neurons		Meurons	
Location	GM, WM, mixed		WM		WM		WM and GM	
Modification	DNA methylation		DNA methylation		DNA methylation		Protein phosphorylation	
Analysis	Bulk BS (mixed 5mC/5hmC)		Bulk BS (mixed 5mC/5hmC)		BS and oxBS (5mC, 5hmC)		IF: P-CREB	

^a^Three individuals (2 MS cases and 1 control) overlapped between cohort 1 and 2, although different brain samples were used, and, eight individuals (5 MS cases and 3 controls) were used for both DNA methylation (cohort 2) and immunofluorescence (cohort IF) analyses, among which 4 brain specimens were identical. *N* number, *F/M* female/male, *PMI* post-mortem interval, *AL* active lesion, *CL* chronic lesion, *CAL* chronic active lesion, *NAWM* normal-appearing white matter, *GM* grey matter, *WM* white matter, *BS* bisulfite, *oxBS* oxidative bisulfite, *5mC* methylation, *5hmC* hydroxymethylation, *P-CREB* phosphorylated CREB, *SD* standard deviation, *IF* immunofluorescence

We next aimed to investigate DNA methylation changes underpinning neuronal vulnerability in MS. Considering the extensive heterogeneity of GM methylome [14], likely due to pervasive phenotypic diversity of GM excitatory neurons [18], and the substantial evidence of WM abnormalities in MS [6–8], we focused on sorted neurons from subcortical WM. We performed DNA methylation analysis on BS-treated DNA isolated from WM-neurons in a first cohort of MS patients and non-neurological controls (cohort 1, *n* = 12, Table 1, Additional file 2) using 450K. After correction for confounders such as sex, age, lesion phenotype, brain region (according to antero-posterior axis) and GLU proportions (the most prominent neuronal subtype within our samples), we detected 13 significant differentially methylated positions (DMPs) between MS and controls (adjusted *P* value < 0.05, Table 2). Gene ontology (GO) analyses of genes associated with the candidate DMPs (unadjusted *P* value < 0.001, Additional file 3) suggested modest enrichment, which did not reach significance after correction for multiple testing, of nervous processes, intracellular signalling pathways and immune/xenobiotic processes (Additional file 4). Notably, some candidate DMP-associated genes map to previously identified differentially methylated regions (e.g. *CREB5*, *DLL1*, *PRKG1*, *PRKCZ* and *DYRK1B* loci) or dysregulated genes (e.g. *SLIT3*, *NOTCH2*, *HLA-DR/-DP*, *GSTT1* or *KCNQ1* genes) in bulk MS NAWM tissue compared to control WM tissue [19] (Additional file 3).

**Table 2:** BS-DMPs (adjusted *P* value < 0.05) associated with multiple sclerosis (cohort 1)

probeID	Chr	Pos	mean_MS	mean_ctr	Δ*β*	*P* value	adj. *P* value	Gene name^a^	Feature^a^
cg25139877	1	247691111	0.90	0.88	0.02	1.02E−06	0.0391	LOC148824	Body
cg19274180	2	2726437	0.62	0.39	0.23	6.79E−08	0.0062	–	–
cg03466083	5	139088813	0.51	0.42	0.08	5.85E−07	0.0273	–	–
cg00211215	6	32552246	0.32	0.23	0.09	1.14E−08	0.0028	HLA-DRB1	Body
cg01414268	8	2480911	0.26	0.20	0.06	1.10E−06	0.0391	–	–
cg10596483	8	143751796	0.35	0.47	− 0.12	7.26E−08	0.0062	JRK	TSS1500
cg27224751	15	41096921	0.53	0.79	− 0.26	8.73E−08	0.0062	DNAJC17	Body
cg14789911	21	47582049	0.65	0.43	0.22	1.25E−06	0.0411	C21orf56	Body
cg03311906	22	20376405	0.77	0.57	0.20	5.23E−07	0.0273	–	–
cg15242686	22	24348715	0.39	0.50	− 0.11	3.45E−07	0.0211	GSTTP1	TSS1500
cg04234412	22	24373322	0.49	0.32	0.17	1.31E−08	0.0028	LOC391322	Body
cg01238044	22	24384105	0.30	0.17	0.13	6.40E−07	0.0273	GSTT1	Body
cg17005068	22	24384525	0.51	0.34	0.18	3.95E−08	0.0056	GSTT1	TSS1500

^a^From UCSC annotations. *Chr* Chromosome, *Pos* position, *MS* multiple sclerosis, *ctr* non-neurological controls, Δ*β* delta *β*-value, *adj. P val* adjusted *P* value, *TSS* transcription starting site

### Separation of true 5mC from 5hmC enables identification of predominant gene body hypo-methylation in MS neurons

In addition to the limited sample size and differences in lesion phenotypes, we hypothesized that heterogeneity in DNA modifications could be an additional contributory factor for the lack of functionally significant changes in the sample set. Indeed, 5hmC is a highly abundant stable modification in the CNS, especially in actively transcribed neuronal genes [20, 21], thus antagonizing 5mC action. Given the inability of BS-based technology to differentiate 5mC from 5hmC, it is highly plausible that specific signals might be lost or misinterpreted depending on the overlapping pattern of 5mC and 5hmC. We further tested this in an additional cohort, focusing on low-grade inflammation WM samples to further reduce heterogeneity.

**Fig. 1:**
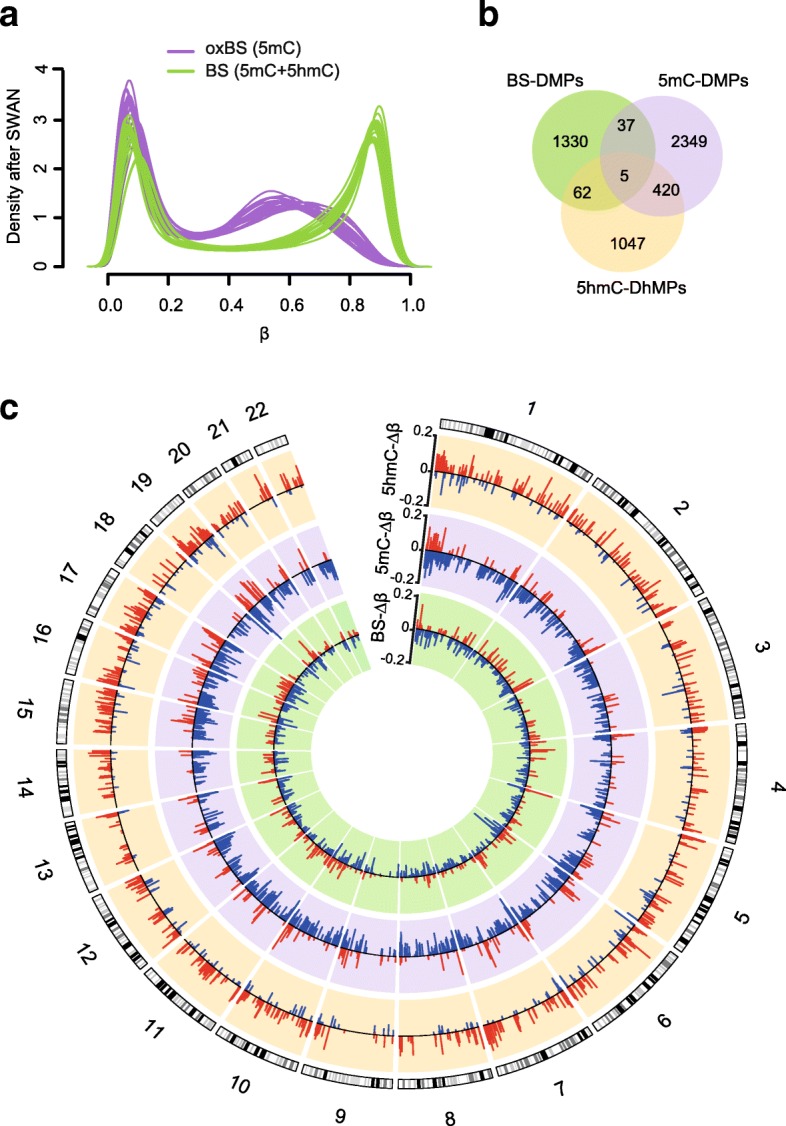
Predominant DNA hypo-methylation and hyper-hydroxymethylation in neurons from multiple sclerosis patients. **a** Density plots of oxBS (purple) and BS (green) *β*-values after subset-quantile within array normalization (SWAN)-based type I/II normalization used for downstream analysis. Venn diagram (**b**) and Circos plot (**c**) illustrating number of significant sites and differences (Δ*β*) in true DNA methylation (5mC, purple), hydroxymethylation (5hmC, orange) and bulk methylation (BS, green) between patients (*n* = 10) and non-neurological controls (*n* = 7) (adjusted *P* value < 0.05). Red and blue circular ideograms represent hyper- and hypo-methylated sites, respectively. The outer track is an hg19 ideogram illustrating chromosome and cytoband information

To examine 5mC and 5hmC changes in MS neurons, we performed traditional BS as well as oxidative BS (oxBS) treatments in parallel, followed by 450K arrays in an additional cohort of MS patients and non-neurological controls (cohort 2, *n* = 17, Table 1, Additional file 2). *β*-values generated by BS conversion represent the total methylation signal (5mC + 5hmC), while *β*-values derived from oxBS conversion reflect only 5mC levels (“true” methylation). Unsupervised hierarchical clustering and MDS showed a clear separation of BS and oxBS samples (Additional file 1: Figure S3). As expected, the corresponding density profiles showed a bimodal distribution of BS and oxBS *β*-values, with oxBS peaking at lower *β*-values (Fig. 1a). This indicates that 5hmC, which is quantified by subtracting corresponding oxBS and BS *β*-value (Δ*β*_BS-oxBS_), accounts for a substantial fraction of the total methylation levels detected in BS samples, and is prominent in sorted neuronal nuclei. In accordance with previous reports [22], 5hmC *β*-values mostly ranged between 0 and 0.5 and peaked at *β*-values ~ 0.25, while a minor fraction of probes displayed slightly negative values (Additional file 1: Figure S4). We therefore applied a stringent stepwise probe-filtering strategy, described in detail in the “Methods” section and summarized in Additional file 1: Figure S5. We could confirm the reliability of our strategy for estimating 5hmC in comparison with the recently published method referred to as OxyBS, which relies on maximum likelihood estimation (oxy-MLE) [23], as 5hmC *β*-values generated by the two methods strongly correlated (Additional file 1: Figure S6).

Of the 419,858 and 272,883 probes used for the subsequent 5mC and 5hmC analyses, respectively, we identified 2811 significant “true” DMPs and 1534 differentially hydroxymethylated positions (DhMPs) between MS and non-neurological controls after correction for confounders (adj. *P* value < 0.05, Fig. 1b, Additional file 5). It is worth noting that 19 DMPs and 19 DhMPS were classified as non-CpG methylation sites. However, the majority of these, 12 and 16, respectively, were located within non-annotated, intergenic regions, therefore not pursued further. The top DMPs and DhMPs (adj. *P* value < 0.01, |Δ*β*| > 0.05) are listed in Table 3. Interestingly, we found a striking predominant hypo-methylation (87%, 2442/2811) and hyper-hydroxymethylation (74%, 1137/1534) in MS patients compared to controls, throughout the genome (Fig. 1c, Additional file 5). Given the high correlation of DNA methylation levels between contiguous CpGs forming co-methylated regions, we sought to identify changes clustering at multiple neighbouring CpG sites, as differentially methylated (DMRs) and hydroxymethylated (DhMRs) regions (Additional file 6). We identified 472 DMRs (5mC-Δ*β* between − 0.27 and 0.13) and 309 DhMRs (5hmC-Δ*β* between − 0.10 and 0.22), the large majority of them (87% of DMRs and 88% of DhMRs) encompassing at least one DMP or DhMP, respectively. Consistent with single CpG analyses, most of the DMRs (88%, 416/472) were hypomethylated, contrasting with the predominant (78%, 243/309) hyper-hydroxymethylation of DhMRs (Additional file 6). We could confirm changes at *OBSCN* locus (chr1: 228503693–228503882) using a BS-free restriction enzyme-based method that distinguishes 5mC from 5hmC (Additional file 1: Figure S7).

**Table 3:** Top 5mC-DMPs, 5hmC-DhMPs and BS-DMPs (adjusted *P* value < 0.05) associated with multiple sclerosis (cohort 2).

probeID	Ch	Pos	mean_MS	mean_ctr	Δ*β*	*P* value	adj. *P* value	Gene name^a^	Feature^a^	Overlap DMP	Overlap DMR
5mC-DMP											
cg06077821	5	180048893	0.50	0.58	− 0.08	7.8E−08	6.6E−03	FLT4	Body	5hmC	5mC, 5hmC
cg04313978	7	158271081	0.60	0.72	− 0.13	6.4E−08	6.6E−03	PTPRN2	Body	5hmC	5mC, 5hmC
cg24172278	7	1526831	0.67	0.75	− 0.08	1.5E−07	8.4E−03	INTS1	Body	–	5mC
cg09226185	7	157342801	0.73	0.81	− 0.08	4.1E−07	9.8E−03	PTPRN2	Body	5hmC	5mC, 5hmC
cg10738865	11	2356706	0.64	0.75	− 0.11	7.2E−10	3.0E−04	–	–	5hmC	5mC, 5hmC
cg12910900	12	13155162	0.62	0.70	− 0.08	2.3E−07	8.9E−03	HTR7P	Body	5hmC	5mC, 5hmC
cg06703573	16	1364331	0.48	0.58	− 0.10	2.6E−08	5.4E−03	UBE2I	Body	5hmC	5mC, 5hmC
cg20942310	17	73728061	0.69	0.78	− 0.08	1.6E−07	8.4E−03	ITGB4	Body	5hmC	5mC, 5hmC
cg15659599	19	55098842	0.36	0.43	− 0.07	4.5E−07	9.8E−03	LILRA2	3′UTR	5hmC	–
cg25934700	22	50644755	0.52	0.66	− 0.14	2.1E−07	8.7E−03	SELO	Body	5hmC	5mC, 5hmC
5hmC-DhMP											
cg18213443	1	4221388	0.21	0.10	0.11	4.7E−07	5.5E−03	–	–	5mC	5hmC
cg27331828	1	196619631	0.27	0.08	0.19	2.9E−07	4.6E−03	CFH	TSS1500	–	5hmC
cg04639610	2	74942001	0.18	0.03	0.15	1.4E−07	4.6E−03	–	–	–	5hmC
cg23376467	5	121464241	0.16	0.05	0.11	2.8E−07	4.6E−03	ZNF474	TSS1500	–	–
cg06996555	7	151542415	0.25	0.12	0.13	1.0E−06	8.3E−03	PRKAG2	Body	5mC	5hmC
cg10738865	11	2356706	0.26	0.15	0.12	1.4E−08	1.5E−03	–	–	5mC	5mC, 5hmC
cg15426626	12	132984261	0.21	0.10	0.11	1.7E−07	4.6E−03	–	–	5mC	5hmC
cg01863290	14	96730669	0.25	0.11	0.13	5.0E−07	5.5E−03	BDKRB1	Body	5mC	5hmC
cg25594736	15	67391147	0.19	0.09	0.10	9.1E−07	8.3E−03	SMAD3	Body	–	–
cg06703573	16	1364331	0.38	0.28	0.11	1.9E−07	4.6E−03	UBE2I	Body	5mC	5mC, 5hmC
BS-DMP											
cg18327319	1	11795409	0.82	0.66	0.17	1.6E−05	2.8E−02	AGTRAP	TSS1500	5hmC	–
cg16732616	1	50886782	0.09	0.21	− 0.12	4.3E−06	2.2E−02	DMRTA2	Body	5hmC	5hmC, BS
cg11750112	1	64938742	0.73	0.83	− 0.10	1.4E−05	2.7E−02	CACHD1	Body	–	–
cg18864124	2	227288863	0.71	0.56	0.14	8.9E−06	2.6E−02	–	–	5hmC	5hmC
cg02379784	5	471326	0.62	0.74	− 0.11	1.0E−05	2.6E−02	LOC25845	Body	–	5hmC, BS
cg08376583	6	32222492	0.33	0.54	− 0.21	8.6E−06	2.6E−02	–	–	–	–
cg25670061	6	163612896	0.77	0.66	0.10	7.2E−06	2.6E−02	PACRG	Body	–	–
cg00459997	12	130621721	0.75	0.63	0.12	2.8E−06	2.2E−02	–	–	5mC	–
cg20165520	13	113637829	0.69	0.57	0.12	1.5E−05	2.7E−02	MCF2L	Body	–	BS
cg05491587	18	77659695	0.59	0.69	− 0.10	6.6E−06	2.5E−02	KCNG2	Body	–	BS

^a^From UCSC annotations. DMP differentially methylated position, DMR differentially methylated region, *5mC* oxBS-generated “true” methylation, *5hmC* hydroxymethylation, *BS* bisulfite-generated, *Ch* Chromosome, *Pos* position, *MS* Multiple Sclerosis, *ctr* non-neurological controls, *Δβ* delta *β*-value, *adj. P value* adjusted P-value, *TSS* transcription starting site

**Fig. 2:**
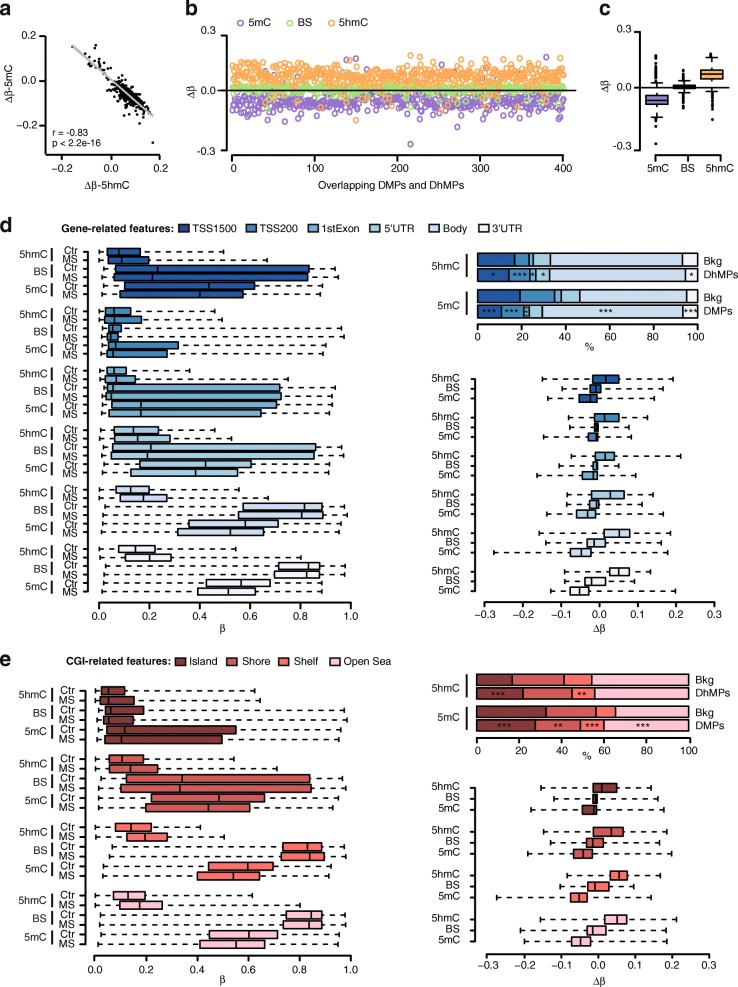
True 5mC and 5hmC changes are anti-correlated and enriched in gene bodies. **a** Spearman’s rank correlation of Δ*β*-5mC (oxBS) and Δ*β*-5hmC at 425 overlapping DMPs/DhMPs (adjusted *P* value < 0.05). Regression line, *P* value (p) and Spearman’s rank correlation coefficient (r) are given. Scatterplots (**b**) and boxplots (**c**) of 5mC (purple), BS (green) and 5hmC (orange) Δ*β*-values at 425 overlapping DMPs/DhMPs (adjusted *P* value < 0.05). Boxplots of *β*-values (left panel), distribution (in comparison to background, bkg) (top right panel) and Δ*β*-values between multiple sclerosis cases (MS, *n* = 10) and non-neurological controls (Ctr, *n* = 7) (bottom right panel) for BS-DMPs, 5mC-DMPs and 5hmC-DhMPs (adjusted *P* value < 0.05) across **d** classical gene features (shades of blue) including promoter-like features (TSS1500, TSS200, 1stExon, 5′UTR), gene body and 3′UTR and **e** CGI-related features (shades of red) including CpG islands, shores, shelves and open sea. In all boxplots, the black line represents the median, the interquartile range is comprised by a box and whiskers are extended to maximum and minimum Δ*β* values. *p < 0.05, **p < 0.01 and ***p < 0.001 using Fisher’s exact test for enrichment and depletion analyses (full data are shown in Additional file 1: Figure S9)

Analyses of BS signals generated 1434 differentially methylated DMPs (BS-DMPs, adjusted *P* value < 0.05) and 193 DMRs (BS-Δ*β* between − 0.23 and 0.21) (Additional files 5 and 6). Notably, only a very small fraction of BS-DMPs overlapped with true DMPs (42/1434) and DhMPs (67/1434) (Fig. 1b), implying that substantial differences between MS patients and controls might be missed or diluted using BS-methodology. Indeed, overlapping DMPs-DhMPs displayed anti-correlated changes (Fig. 2a), resulting in unchanged BS-Δ*β* values for these sites (Fig. 2b, c). This strongly suggests that opposing 5mC and 5hmC levels cancel out differences in the conventional BS signal, and that investigating 5mC and 5hmC alterations separately allows the discovery of a substantial fraction of changes occurring in neurons. Accordingly, we found a strong positive correlation (*P* value < 2.2.10^−16^, *r* = 0.73) between oxBS-Δ*β* (adjusted *P* value < 0.05) and BS-Δ*β* (*P* value < 0.001) at overlapping sites, and, to a lesser extent (*P* value < 2.2.10^−16^, *r* = 0.33) at all sites present in the array (Additional file 1: Figure S8). Collectively, these findings suggest that many BS changes with *P* value < 0.001 are likely true and that the sensitivity for detection of significant changes using BS data could be significantly compromised by dilution with 5hmC signals.

As previously mentioned, 5hmC has been shown to display a unique genomic distribution compared to 5mC and to exert antagonistic roles on regulation of transcription [15]. We stratified 5mC and 5hmC changes based on gene features annotated in 450K and found overall hypo-methylation and hyper-hydroxymethylation in MS patients compared to controls across all gene features (Fig. 2d). The largest 5mC and 5hmC changes occurred within the gene body and 3′UTR, with a smaller contribution from regions harbouring promoter-like features (TSS1500, TSS200, 5′UTRs and 1^st^ exon) (Fig. 2d). Fisher’s exact test revealed that 5mC-DMPs are strongly enriched (*P* value = 1.16 × 10^−39^) within gene bodies while depleted (*P* value = 4.24 × 10^−23^ for TSS1500) from segments with promoter-like features (Fig. 2d, Additional file 1: Figure S9). Finally, analysis in relation to CpG islands (CGIs) indicated that CGIs displayed the lowest 5mC, 5hmC *β*-values and |Δ*β*|-values compared to shores and shelves (Fig. 2e). We found enrichment of 5hmC-DhMPs in CGIs (*P* value = 1.85 × 10^−07^), which are depleted (*P* value = 3.27 × 10^−09^) of 5mC-DMPs (Fig. 2e, Additional file 1: Figure S9). Of note, the backgrounds used for 5mC and 5hmC analyses are noticeably different.

Thus, all explored gene features generally showed evidence of negatively correlated changes in 5mC and 5hmC, with DMPs and DhMPs predominantly enriched in gene bodies and CpG islands, respectively.

### 5mC and 5hmC changes of MS neurons associate with genes involved in CREB signalling pathway, synaptic plasticity and axonal guidance

**Fig. 3:**
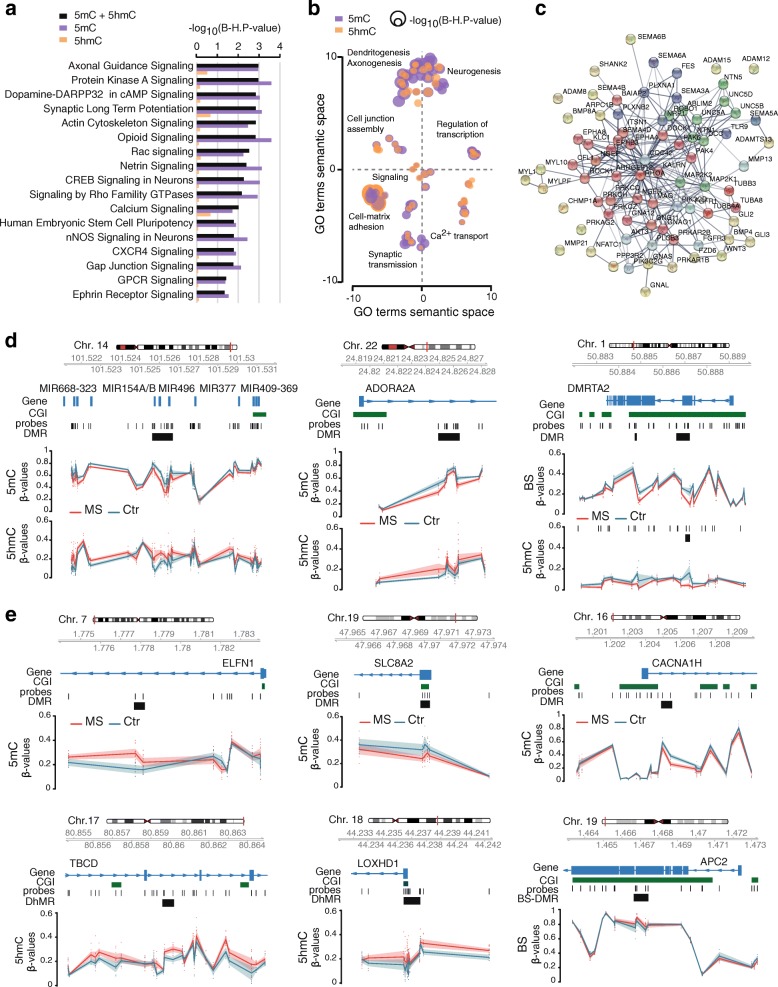
Functional association of 5mC and 5hmC changes to axonal guidance, CREB signalling and synaptic plasticity. **a** Top canonical pathways associated with “true” methylation (5mC) and hydroxymethylation (5hmC) between multiple sclerosis cases (MS, *n* = 10) and non-neurological controls (Ctr, *n* = 7) using Ingenuity Pathway Analysis (IPA). Results for all adjusted *P* value < 0.05 5mC-DMPs +5hmC-DhMPs (black) as well as 5mC-DMPs (purple) and 5hmC-DhMPs (orange) separately. Significance is represented as − log_10_(B-H, *P* value) after adjustment using Benjamini-Hochberg (B-H) correction. **b** Multidimensional scaling of over-represented biological function terms associated with 5mC-DMPs (purple) and 5hmC-DhMPs (orange) (adjusted *P* value < 0.05), separately, between MS cases (*n* = 10) and Ctr (*n* = 7), according to semantic similarities, using REVIGO. The circle size represents − log_10_(B-H, *P* value) after adjustment using Benjamini-Hochberg (B-H) correction. **c** Representation of the genes from axonal guidance network using STRING analysis. Grey gradient indicates the strength of data support (darker grey representing stronger evidence) and colours represent different cluster (kmeans clustering set at 5). **d** Plots illustrating genes associated with top 5mC-DMR and 5hmC-DhMR (left and middle panel) or with BS-DMRs and 5hmC-DhMRs (right panel) (|Δ*β*| > 0.05). **e** Plots illustrating genes associated with top 5mC-DMR, 5hmC-DhMR or BS-DMRs (Δ*β* > 0.05). The hg19 ideogram illustrating chromosome and cytoband information, the complete gene structure of the locus (blue track), CpG Island (CGI) feature (green track), probes and DMR (black) location are shown. Methylation (*β*-values) of single CpGs within and outside the DMR for individual cases and controls is depicted in red and blue, respectively, with connecting lines indicating mean methylation for each consecutive CpG

To gain insight into biological functions associated with changes in 5mC and 5hmC, we performed ingenuity pathway analysis (IPA) on 2448 genes associated to all significant sites, i.e. 2811 DMPs + 1534 DhMPs (adj. *P* value < 0.05). Top enriched pathways associated to D(h) MPs remained when relaxing the significance of CpG sites (unadjusted *P* value < 0.001), and reflected changes in both 5mC and 5hmC (Fig. 3a, b Additional file 4). The most significant canonical pathways were related to nervous system processes, particularly axonal guidance (Fig. 3c), cAMP response element-binding (CREB) signalling and synaptic plasticity, to other intracellular signalling pathways such as actin cytoskeleton signalling and to oxidative stress/inflammation processes (Fig. 3a, b, Additional file 4). IPA analysis of BS-DMPs showed modest enrichment for intracellular signalling pathways such as xenobiotic metabolism and integrin signalling (Additional file 4). GO findings were confirmed when focusing at region levels where DMRs + DhMRs were enriched in genes implicated in regulation of neuron projections (e.g. *PLXN4A*, *NTN1/5*, *RHOT1/2*, *CYFIP1*, *DOCK1*, *KIF1A*, *PACSIN1*, *RIN1* genes), neuronal development (e.g. *CFL1*, *ISL1*, *ADAP1*, *DIABLO*, *PTK2B*, *MYBL2* and *TP73*), as well as ion channels (e.g. *KCNQ1*, *KCNAB2*), and GABA/glutamate activity (e.g. *GABBR1*, *GRIN2D*, *LRP1*, *ABAT*) (Additional file 4). Examples of dendritic and axonal (*TBCD*, *APC2*), synaptic (*ADORA2*, *ELFN1*, *SLC8A2*, *CACN1H*) and neurogenic (*DMRTA2*, *LOXHD1*) DMR-associated genes are illustrated in Fig. 3d and e. Findings were further supported by GO analysis of the predicted target genes from differentially methylated miRNAs identified in cohort 2 (exemplified in Fig. 3d, Additional file 4), axonal guidance being the most enriched pathway (Additional file 4).

**Fig. 4:**
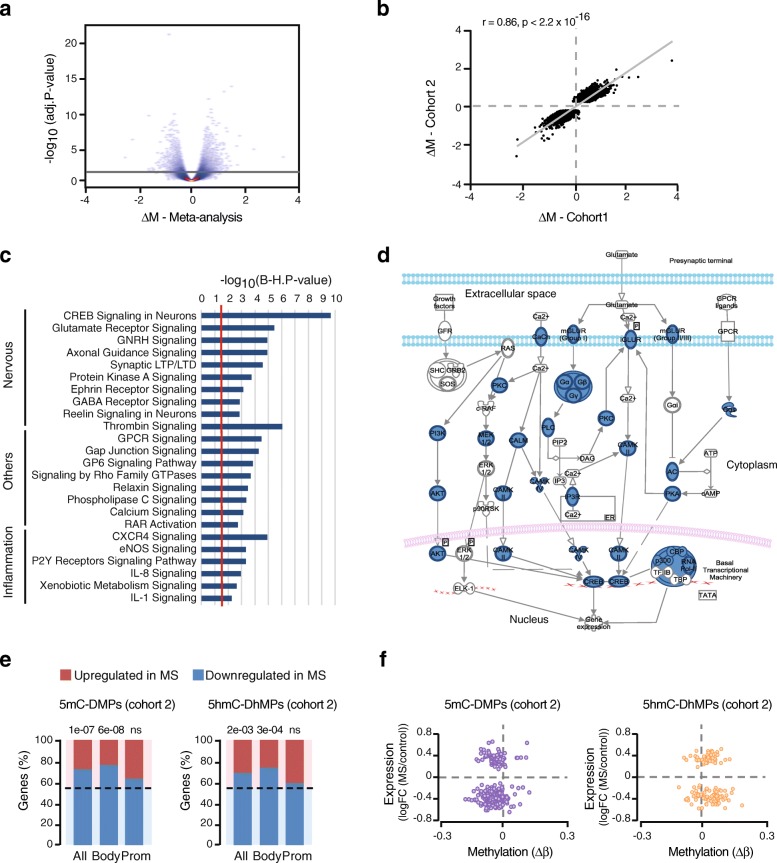
Meta-analysis of cohorts and comparison of DNA methylation with transcriptional changes. **a** Volcano plot illustrating differences in *M*-values between multiple sclerosis (MS) cases and non-neurological controls (adjusted *P* value < 0.05) from meta-analysis of cohorts 1 and 2. **b** Correlation between effect sizes (Δ*M*) of cohorts 1 and 2 for DMPs identified in meta-analysis (adjusted *P* value < 0.05) revealed predominant same direction of changes in the two cohorts. Regression line, *P* value (p) and Spearman’s rank correlation coefficient (r) are given. **c** Top canonical pathways of DMPs (adjusted *P* value < 0.05) identified in meta-analysis. Significance is represented as − log_10_(B-H, *p* value) with the red line showing threshold of significance after adjustment using Benjamini-Hochberg (B-H) correction. **d** Schematic representation of CREB-signalling pathway associated with DMPs from meta-analysis, with differentially methylated genes between cases and controls depicted in blue. **e** Association of 5mC-DMPs (left) and 5hmC-DhMPs (right) with gene expression data (RNA-seq) in bulk NAWM vs control WM [19]. Proportions (percentage) of number of upregulated and downregulated genes are depicted in red and blue, respectively (the dotted line represents the expected proportion). *P* values (p) generated with the Chi-square test are given for DMPs located in gene body or promoter-associated features (TSS1500, TSS200, 5′UTR and 1stExon) (full data in Additional file 8). **f** Scatter plots illustrating association between 5mC-DMPs (purple, left) and 5hmC-DhMPs (orange, right) methylation changes (Δ*β*-value) from cohort 2 (adjusted *P* value < 0.05) with gene expression data (RNA-seq) in bulk NAWM vs control WM [19]. The number of samples used in cohorts 1 and 2 equals to *n* = 12 and *n* = 17, respectively

Considering the limited sample size, we aimed to validate pathways and functions by performing a meta-analysis of the BS data generated for both cohorts. We took advantage of the strong correlation identified between true 5mC and BS-generated DNA methylation changes (Additional file 1: Figure S8). A total of 265,129 common BS-derived probes considered homogeneous between the two studies (*I*^2^ < 15%, Additional file 1: Figure S10) were examined. Meta-analysis identified 8281 positions (Benjamini-Hochberg adjusted *P* value < 0.05, Fig. 4a, Additional file 7), the large majority (97%) exhibiting same direction of changes in both cohorts (Fig. 4b, Additional file 1: Figure S10). Pathway investigation of the genes associated with DMPs confirmed strong enrichment of CREB signalling in neurons and associated pathways, followed by other nervous system processes (e.g. axonal guidance), intracellular signalling pathways and inflammatory/oxidative processes (Fig. 4c, Additional file 4). Accordingly, altered genes are predominantly involved in CREB signalling (Fig. 4d) and comprise several subunits of the ionotropic NMDA (*GRIN1*, *GRIN2A-C* genes), AMPA (*GRIA4*), delta (*GRID2*) and kainate (*GRIK3-4* genes) and metabotropic (e.g. *GRIM1*, *GRM3-4*) glutamate receptors as well GABA receptors (e.g. *GABBR1*, *GABRA1*, *GABRB3*, *GABRG2*). Downstream pathways involve multiple signalling molecules such as PKA and PKC genes, downstream cAMP-dependent kinases (*CAMK2/4* genes) and transcriptional regulators (*CREBBP*, *CREB5*, *EP300*), among others. Axonal guidance genes are associated with ephrin/Eph receptors (e.g. *EFN3-5*, *EPHA1*, *EPHA2*, *EPHA4*, *EPHB1*, *EPHB6*) and semaphorin/plexin (e.g. *SEMA4A*, *SEMA6A-C*, *PLXNA2*, *PLXND1*) with Slit/ROBO (*SLIT1/3*, *ROBO1/2*) complexes together with actin-cytoskeleton molecules as well as trophic factors (*BDNF*, *NGF*) and downstream TGF-*β*, Shh and Wnt signalling pathways (e.g. *BMP4*, *WNT5A*, *GLI2*). Finally, inflammatory processes include cytokine signalling (e.g. *TNF*, *IL1RAP*, *IRAK3*, *TRAF6*, *MAP2K4*, *MAP3K1*, *MAPK11*), oxidative pathways (e.g. *NOS3*, *SOD3*, *NOSTRIN*, *HSPA1A/B*, *HSP90B1*) and xenobiotic metabolism such as the aryl hydrocarbon receptor (*AHR*, *AHRR*), glutathione- and cytochrome P450-mediated detoxification enzymes (e.g. *GSTM1/3/5*) (Additional file 4).

We then investigated the putative functional impact of DNA methylation changes by examining corresponding differentially expressed (DE) genes reported in RNAseq data from bulk MS-NAWM compared to control WM [19]. Of 4669 genes associated with 8281 DMPs from the meta-analysis, 84.4% were detected in the RNAseq analysis, among which 13% (510) were differentially expressed (unadjusted *P* value < 0.05). Of those, 61% (312/510) were significantly downregulated in bulk MS NAWM compared to control WM [19] (Additional file 8). Interestingly, genes harbouring methylation changes within gene body showed the largest proportion of downregulated genes (72%), which is significantly more than expected by chance (*p* = 4.5 × 10^−13^, Chi-square test), as opposed to changes within region of promoter-like features (54%) (Additional file 8). A predominant downregulation was also evident for true 5mC and 5hmC (cohort 2) (Fig. 4e,f), with for example 76% (129/170) of genes affected by gene body 5mC-DMPs displaying a significant decreased expression in bulk NAWM compared to WM (*p* = 6.0 × 10^−08^, Chi-square test, Additional file 8). This suggests that gene body hypo-methylation is likely associated with decreased transcriptional activity, as previously suggested [24, 25].

Altogether, these data strongly suggest that WM-neurons from MS patients manifest 5mC and 5hmC differences that might impair neuronal homeostatic functions, possibly through transcriptional regulation of associated genes. Most of the changes converge on alteration of CREB signalling pathway, synaptic plasticity and axonal guidance.

### DNA methylation changes associate with reduced CREB activity in NAWM neurons

**Fig. 5:**
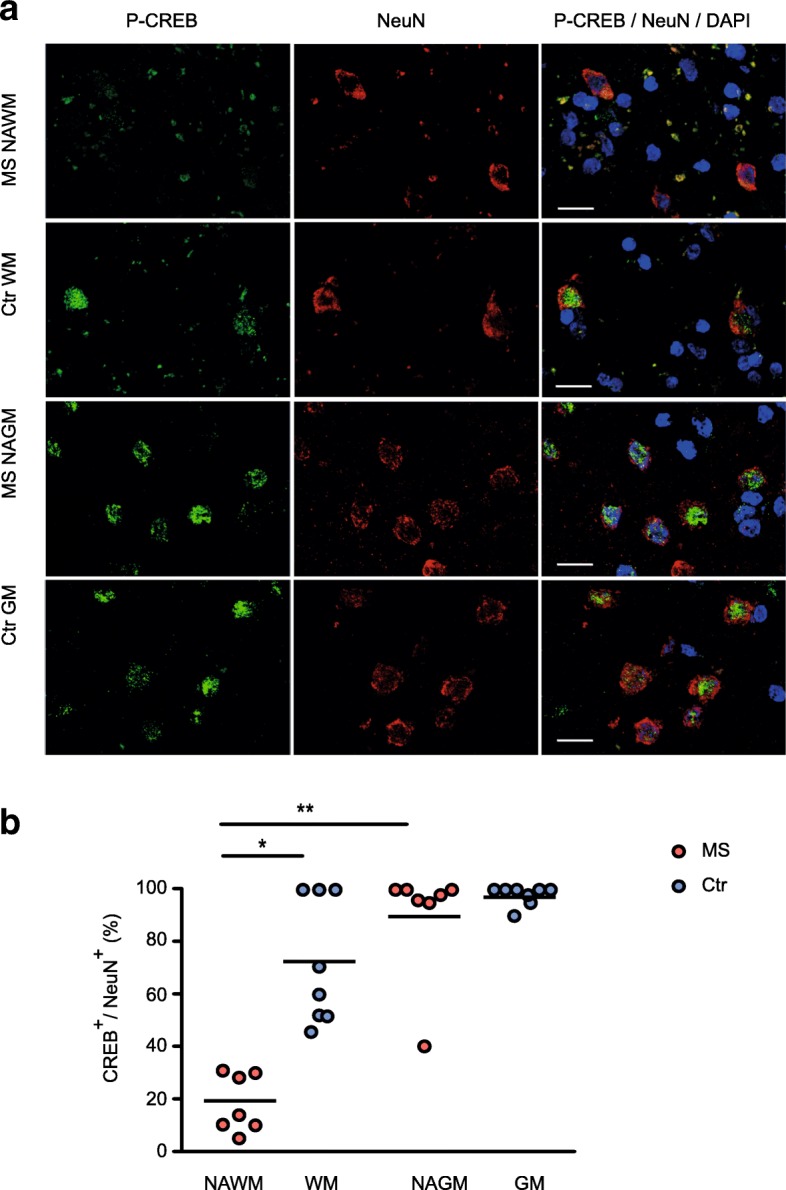
Reduction of CREB activity in normal-appearing white matter neurons. **a** Representative images of multiple sclerosis (MS) and non-neurological control (Ctr) brain tissue sections co-targeted with phosphorylated CREB (P-CREB, green, left panel) and neuronal marker NeuN (red, middle panel); merged with DAPI (blue, right panel) assessed by immunofluorescence (IF) (magnification 630x, scale bar 15 μm). **b** Quantification of IF images from MS cases (*n* = 7) and Ctr (*n* = 8). The amount of NeuN^+^/CREB (phospho S133)^+^-neurons is presented as a percentage of the total amount of all detectable NeuN^+^ cells. In contrast to NAWM, CREB activation status in the NAGM neurons was less affected and comparable between the groups. *p < 0.05, *p < 0.01 using Kruskal-Wallis test and Dunn’s test for multiple comparisons. NAWM, normal-appearing white matter, NAGM, normal-appearing grey matter, WM, White matter, GM, Grey Matter

DNA methylation analysis identified alteration of multiple genes involved in CREB response in MS neurons, some of which were dysregulated in transcriptome or methylome studies of heterogeneous MS brain tissue [19] (Additional file 7). We then asked whether DNA methylation changes in CREB-related genes could associate with alteration of CREB activation in neurons from MS patients. We addressed this by examining the activation status of CREB transcription factor using immunofluorescence (IF), reflected by nuclear phosphorylated CREB (P-CREB^+^) in MS and non-neurological controls (*n* = 15, a cohort overlapping cohort 2, Table 1, Additional file 2). Group-representative images are shown in Fig. 5a. Analysis revealed a significantly lower number of neurons with nuclear phosphorylated CREB (P-CREB^+^) in the NAWM of MS patients (18.3 ± 11.0%) compared to controls (72.5 ± 23.9%) (*p* = 3.4 × 10^−02^, Kruskal-Wallis with Dunn’s multiple comparisons test, Fig. 5b). In contrast, no significant change in the activation status of CREB could be detected in normal-appearing GM from MS patients compared to control GM (Fig. 5b), implying that NAWM neurons of the MS brain seem to be more susceptible to dysfunction of the CREB signalling pathway than the GM ones.

## Discussion

We here utilized DNA modifications to investigate processes that occur in neurons from MS patients. To our knowledge, this is the first report of 5mC and 5hmC abnormalities in neurons, especially WM-neurons, in the context of neurological disease. We found striking hypo-methylation and hyper-hydroxymethylation at specific CpGs, some of which clustered into regions and occurring mainly within gene bodies. Our findings implicate alterations of genes involved in axonal guidance, synaptic plasticity and CREB signalling, as putative key processes that could contribute to impaired neuro-axonal integrity and inability to repair after immune insult, and perhaps also be more prone to degenerate. Importantly, we could associate DNA methylation changes with functional outcomes by reporting for the first time a reduction of CREB activity in NAWM neurons compared to control WM neurons.

Our data show opposing hypo-5mC and hyper-5hmC changes, which seem to co-localize mostly within gene bodies. Additionally, we observed that a large majority of genes acquiring gene body hypo-5mC/hyper-5hmC was significantly downregulated in bulk MS-NAWM vs. control WM [19], linking these DNA modifications with transcriptional activity. Several studies have shown that hypo-5mC in post-mitotic neurons associates with impaired survival and excitability [26–28]. Likewise, hyper-5hmC can lead to neuronal hypersensitivity [29] and defects in neurite outgrowth and synaptic formation[30]. Moreover, a growing body of evidence suggests that global hypo-5mC and hyper-5hmC changes result in subsequent TET-mediated activation of specific endogenous retroviral (ERV) elements [31, 32], that can ultimately result in severe postnatal neurodegeneration in rodents [31]. Importantly, strong transcriptional activity of human endogenous retroviruses, including in CNS cells, has been detected in MS patients [33]. Thus, altered 5mC and 5hmC patterns might contribute to neuronal vulnerability by impairing chromatin regulatory architecture in MS neurons, leading to dysregulation of neuron-specific genes and enhanced genome instability.

Whereas specific mechanisms affecting levels of 5mC and 5hmC in MS neurons remain to be explored, several processes such as oxidative stress, inflammation or hypoxia together with putative upstream regulators identified in our study (e.g. TGF-*β*1, POU5F1, CREB1, Additional file 4), may impact locus-specific 5mC and 5hmC profiles. Moreover, an emerging body of evidence suggests a putative role of dysregulated epigenetic enzymes such as DNMT, TET and MBD genes in MS, as observed in blood cells [34] and brain [35] from MS patients compared to controls. This is further supported by a recent study showing reduced methionine levels in plasma of MS patients and the regulatory effect of peripheral methionine on DNMT3A in the mouse brain [36]. Consistent with this, we found that several key DNA methylation enzymes, namely DNMT1, DNMT3A, DNMT3B or MBD3, among others, displayed significant 5mC and/or 5hmC (cohort 2) and BS (meta-analysis) DNA methylation changes in MS neurons compared to controls. Finally, since DNA methylation levels can be affected by variations in DNA sequence, it may be speculated whether MS risk alleles such as that on chromosome 4 encompassing the TET2 gene [37], might contribute to the observed changes in 5mC and 5hmC levels. In line with this, TET2 promoter exhibited reduced 5mC levels in MS neurons (cohort 2). Thus, the combined influence of neuroinflammatory processes occurring as a result of MS as well as putative upstream MS disease risk factors may dually impact on 5mC and 5hmC profiles in MS neurons.

While axonal injury is believed to be a major contributor to disability such as cognitive decline in late MS [6–8], comprehensive characterization of the underlying mechanisms is still lacking. Moreover, accumulating evidence points to a role of glutamate excitotoxicity [38, 39] and synaptopathy [40, 41] in neuro-axonal dysfunction and degeneration in MS. Importantly, glutamate levels measured in NAWM of MS patients are predictive of neuro-axonal integrity, brain atrophy and cognitive impairment [42, 43]. In our study, neurons from MS patients displayed epigenetic alterations affecting several genes of the glutamate/GABA signalling, including multiple subunits of glutamate and GABA receptors. Additionally, our data suggest that compromised axonal architecture likely results from changes in interconnected cellular networks ranging from surface complexes (semaphorin/plexin, Ephrin, Slit/ROBO) to genes from the cytoskeleton and Shh/Wnt-signalling pathways. Comparison of samples from the same individual containing different lesion phenotypes (pilot samples, Additional file 1: Figure S1) suggests lesion-associated changes in genes implicated in neuronal projections (e.g. *EPHA10*, *NIN*, *ALCAM*) and synaptic processes (e.g. *GABRA5*, *PRKG1*, *DLGAP3/SAPAP3*) as well. Thus, neurons of MS patients exhibit epigenetic alterations that likely reflect neuro-axonal damage and/or failure in compensatory mechanisms.

Interestingly, DNA methylation changes converged on genes in the CREB signalling pathway, namely PKA, PKCs, CAMKs kinases and CREB genes. Transcription factors of the CREB family play pivotal roles in axonal regeneration, plasticity, cell survival, oxidative stress and neuroprotection [44, 45]. Prior studies have reported dysregulated CREB signalling in lesions from bulk/mixed MS brain tissue [40, 46]. Importantly, we could associate DNA methylation differences with reduction of CREB phosphorylation in NAWM neurons from MS patients compared to controls, which is to our knowledge the first report of altered CREB activity in NAWM in MS. Undeniably, one cannot exclude additional mechanisms affecting CREB activity independently of DNA methylation changes. Nevertheless, this is highly relevant considering the CREB-mediated neuroprotective effect exerted by the MS drug fingolimod (Gilenya™; Novartis, Basel, Switzerland) in vitro and in vivo in a model of neurodegenerative motor disease [47]. Interestingly, CREB activation differed between WM and GM neurons, likely reflecting differences in neuronal subtypes and/or distinct susceptibility towards demyelination pathology [48], such as hypoxia-like metabolic injury [49]. Altogether, these findings strongly suggest that epigenetic and functional alteration of CREB signalling can occur without or prior to focal tissue damage.

Undoubtedly, epigenetic marks are critical features of cellular differentiation and phenotype but multiple confounders could bias proper interpretation of DNA methylation data. Therefore, we aimed to minimize tissue- and DNA modification-driven sources of heterogeneity by studying neuronal nuclei isolated from WM, correcting for neuronal subtype proportions and separating true 5mC from 5hmC. To our knowledge, this strategy has not been previously reported in a neuropathological context. Our results suggest that BS-based DNA methylation studies might preferentially capture strong difference in one epigenetic mark and that examining 5mC and 5hmC separately could greatly aid in detecting novel changes that might have escaped detection by conventional BS-based DNA methylation studies. Nevertheless, in our study, BS-DMP changes appear strongly correlated with 5mC differences. Moreover, some of our findings could replicate previously reported DNA methylation results and could further associate with differential gene expression in bulk NAWM compared to control WM [19]. The aberrations in WM-neurons of MS patients identified here are interesting in light of neuronal circuit disconnection [50] and subsequent disability in progressive MS. Additional cell type-specific studies in larger cohorts can shed further light on mechanisms underlying neuronal dysfunction and associated disability. In the long term, a better understanding of functional implications of epigenetic changes in CNS cells in MS may lead to novel therapeutic strategies aiming at restoring neuronal integrity and ameliorating cognitive deficit in progressive MS [51, 52].

## Conclusions

Our study demonstrates functionally relevant DNA methylation alterations in WM-neurons from MS patients in comparison to non-neurological controls, thereby providing new insights into mechanisms underlying neuro-axonal pathology of the WM and clinical symptoms in MS patients. Furthermore, our findings open new perspectives for similar approaches based on deciphering true 5mC and 5hmC changes in neurons specifically in other neurological disorders.

## Methods

### Subjects and cohorts

Brain tissues of all the cohorts was obtained from the Multiple Sclerosis and Parkinson’s Tissue Bank (Imperial College London). Briefly, the pilot samples were used to assess the impact of sample heterogeneity on DNA methylation profiles, cohorts 1 and 2 were used to analyse neuron-specific DNA methylation changes between MS cases and controls and cohort-IF overlapping with cohort 2 was utilized for functional validation of CREB activity in brain sections. Further sample details are given in Additional file 2. The material comprises snap-frozen brain tissue blocks collected within 33 h post-mortem and divided by specialist into histopathologically characterized lesion categories: active lesion (AL), chronic active lesion (CAL), chronic inactive lesion (CL) and normal-appearing white matter (NAWM). Control subjects were selected based on a non-neurological cause of death. The samples were further annotated for cerebral location (antero-posterior axis), characteristic of the tissue (mixed, white or grey matter) using the human brain atlas sectional anatomy database (http://www.thehumanbrain.info/) and were further dissected accordingly. The anatomical localization of the samples across brain regions is illustrated in Additional file 1: Figure S11. Of note, different brain samples from same individuals were used between cohort 1 and 2, and, four identical specimens derived from eight individuals were used for DNA methylation study (cohort 2) and functional validation. No formal sample size calculation was conducted, samples reaching the following inclusion criteria have been included in DNA methylation analyses: (1) all available samples with sufficient DNA amount, (2) samples that passed DNA methylation quality control and (3) cases with confirmed MS diagnosis and non-neurological controls without any signs of inflammation in the CNS. The pilot samples were selected based on the availability of different lesion phenotypes within the brain of one MS patient. Cohort 1 was selected based on available amount of DNA from WM neuronal nuclei. Cohort 2 was selected based on sufficient DNA amount from WM neuronal nuclei to perform both 5mC and 5hmC analyses and based on the low-grade inflammation of the lesion phenotype with only data from WM-neuronal nuclei being used for the comparison MS versus non-neurological control. Cohort used for functional validation was selected based on the availability and quality of the material.

### Sample preparation

Fluorescence-activated cell sorting (FACS)-based neuronal nuclei isolation from dissected brain tissue was performed according to previously published protocol [53] (see representative experiment in Additional file 1: Figure S1). Briefly, following resuspension of the homogenized brain tissue in hypotonic lysis buffer (0.32 M sucrose, 5 mM CaCl_2_, 3 mM MgAc_2_, 0.1 mM EDTA, 10 mM Tris pH.8, 1 mM DTT, 0.1 % Triton), nuclei were extracted by ultracentrifugation in sucrose gradient (1.8 M sucrose, 3 mM MgAc_2_, 1 mM DTT, 10 mM Tris pH.8) for 2.5 hours at 4 °C. Nuclei were further labelled with Alexa Fluor 488 (Invitrogen #A11029)-conjugated anti-NeuN antibodies (1:700, Millipore #MAB377) and separated into neuronal and non-neuronal nuclei by flow cytometry (MoFlo^TM^ high-speed cell sorter). First, FSC [Par] × SSC gating was used to separate larger particles from smaller debris. Next, FSC [Par] × Trigger Pulse Width plot was used to remove aggregated nuclei such as duplicates. The fluorescence event plot showed two clear populations including the NeuN-positive fraction (representing 4–40% of the nuclei). We confirmed that positive fractions were negligible in negative controls, i.e. no antibody or unconjugated Alexa Fluor 488-labelled nuclei. Neuronal nuclei were pelleted and stored at − 80 °C until DNA isolation. Genomic DNA was isolated using QIAmp DNA micro kit (QIAGEN), resuspended in water and stored at − 80 °C.

### Illumina Human Methylation 450K

We used Illumina Infinium Human Methylation 450K BeadChip (Illumina, Inc., San Diego, CA, USA; 450K) for quantitative and genome-wide DNA (hydroxy) methylation profiling. Genomic DNA was subjected to either conventional BS-treatment for the pilot samples and cohort 1 or BS and oxidative BS (oxBS)-conversion using TrueMethyl^TM^ 96 kit of CEGX^TM^ (Cambridge Epigenetix Limited) for cohort 2. BS-DNA from the pilot samples and cohort 1 was hybridized to 450K arrays at BEA core facility (Karolinska Institutet), oxBS/BS-DNA from cohort 2 were processed at GenomeScan (GenomeScan B.V., Leiden, The Netherlands), according to manufacturer’s instructions and the BeadChip images were scanned on the iScan system. Samples were randomized ensuring that disease group, gender and age were balanced to control for potential confounding effects. Technicians performing 450K arrays were blinded to the MS disease status during the experiments. Persons performing statistical analysis were not blinded to disease status. The analysts have never altered the diagnosis of samples and no individuals were excluded because of diagnosis.

### DNA methylation and hydroxymethylation analyses

#### Quality control

450K data (485,577 probes) were quality assessed using MethylAid [54], which examines Red(R)/Green(G) signal intensity, bisulfite conversion, specificity, staining, extension, target removal and hybridization as well as overall performance of the assay. All samples passed quality control and were subsequently processed using the Chip Analysis Methylation Pipeline (ChAMP) version 1.8.0 [55] and minfi version 1.16.0 [56] Bioconductor packages.

#### Probe filter

Upon loading raw IDAT files into ChAMP, probes were filtered by detection *P* value > 0.01, bead count < 3 in at least 5% of the samples, SNPs (minor allele frequency > 1% in the European population) and cross-reactivity as identified by Nordlund et al. [57] (pilot study) and Chen et al. [58] (cohorts 1 and 2). After filtering, the remaining probes for the pilot samples, cohort 1 and cohort 2 reached 374,756, 427,712 and 419,958, respectively. Notably, probes located on the X and Y chromosomes were also removed, as samples from both females and males were included in the study.

#### Between and within-array normalization

*β*-values of remaining probes were either between-sample quantile normalized followed by within-sample Beta-mixture quantile normalization (BMIQ)[59] as previously recommended [60] in ChAMP for the pilot study or within-sample normalized using the “Subset-quantile Within Array Normalization” (SWAN) method[61] as previously recommended for oxBS-450K data [22] in minfi for cohorts 1 and 2. Noticeably, within-sample normalization corrects for two different probe designs (type I and type II probes) included on the 450K BeadChip.

#### Hydroxymethylation (5hmC)

Filtering strategy and pipeline workflows are illustrated in Additional file 1: Figures S4 and S5. The champ. TrueMethyl function (ChAMP Bioconductor package version 1.8.0) [55] was applied to SWAN-normalized *β*-values to identify the “most variable positions” between oxBS and BS samples. The default Benjamini-Hochberg cut-off (B-H. adj. *P* value < 0.05) was used and filtered 110,666 sites, which predominantly encountered probes with mean hydroxymethylation levels around 0. Negative average hydroxymethylation sites (3383 probes, ~ 1%), were considered false positives and therefore also removed. *P* value distributions confirmed that mean 5hmC values > 0 had lower *P* values than mean 5hmC values < 0 (Additional file 1: Figure S4). Of the remaining 305,809 probes, 5hmC *β*-values were calculated by subtracting BS and oxBS *β*-values. Probes with > 1 negative value (28,421 probes) were filtered and since slide 4 only contained 2 neuronal nuclei DNA samples, no negative 5hmC values were allowed on slide 4 (causing 4505 probes to be removed) to allow for subsequent batch/slide correction with ComBat [62]. For comparison, we tested another method based on maximum likelihood estimation (MLE) available through the oxyBS version 1.0 Cran package [55] with default settings.

#### Correction for slide-effect

Slide effects were corrected using empirical Bayes methods [62] implemented in the ComBat function of the SVA Bioconductor package (version 3.18.0). Principal component analysis (PCA), combined with cofactor association testing before and after ComBat, confirmed that the slide-effect was successfully removed.

#### Neuronal subtypes deconvolution

DNA methylation sites previously identified to significantly differ between GABA and GLU (FDR < 0.05) were retrieved from [17] and filtered for |Δ*β*| > 0.7 which resulted in a total of 162 neuronal subtype-specific sites. In concordance with previous observations [17], the majority of the 162 examined neuronal subtype-specific CpG sites, were hypomethylated in GLU compared to GABA neurons (Additional file 1: Figure S2). Notably, due to probe filter (for details see above), the number of cell type specific probes was reduced to 144 for cohort 1 and 143 for cohort 2, respectively. GABA and GLU cell proportions were estimated from raw BS *β*-values using Houseman’s reference-based algorithm [63] as implemented in the projectCellType() function of the minfi Bioconductor package [56]. Estimated cell proportions were confirmed using the robust partial correlations (RPC) method from the EpiDISH (*Epi*genetic *D*issection of *I*ntra-*S*ample *H*eterogeneity) R package version 1.0.0 [64] (Additional file 1: Figure S2). While we found no significant differences in GABA and GLU neuronal proportions between MS and non-MS controls in neither cohorts (Additional file 1: Figure S2), Spearman’s rank correlation analysis revealed correlation with top PCs (Additional file 1: Figure S2), indicating that GLU proportions (the most prominent neuronal subtype in our samples) contribute to variation of DNA methylation in our samples. Sensitivity modestly increased after including GLU proportions as a co-variate in the linear model (Additional file 1: Figure S2). Heat maps and scatter plots of GABA and GLU specific probes were generated using the “ComplexHeatmap” Bioconductor package version 1.17.1 [65]. Putative differences in cell proportions between MS and non-MS controls were assessed with non-paired *t* tests and visualized with the ggboxplot() function of version 0.1.6 ggpubr R package. Venn diagrams were generated using the draw.pairwise.venn() function of version 1.6.20 VennDiagram R package.

#### Differentially methylated positions (DMPs) and regions (DMRs)

The Limma Bioconductor package version 3.26.3 [66] was used for detection of DMPs with *M*-values (*M*_i_ = log2(*β*_i_/(1 − *β*_i_))) as input as previously recommended [67]. The following covariates, as confirmed by PCA, were included in the model: Individual, Sex, Age, Lesion phenotype, Brain localization (according to antero-posterior axis) and GLU proportion for cohort 1 and Sex, Age, Lesion phenotype, Brain localization and GLU proportion for cohort 2. The influence of brain regionality has been investigated using association analysis, covariate regression and randomization and potential confounding effects of brain regionality have been excluded. DMRcate version 1.6.53 [68], which identifies DMRs based on kernel smoothing, was applied with default settings (*λ* = 1000, *C* = 2).

#### Meta-analysis

Meta-analysis of cohort 1 and 2 BS-derived 414,306 common probes was conducted using the inverse variance based method of the METAL tool, which weights the effect size for each study by their standard error [69]. Effect size estimates were retrieved as logFC (*M*-value based) and standard error estimates as sqrt (fit$s2.post) × fit$stdev.unscaled from Limma outputs, respectively. Simultaneous heterogeneity testing allowed for subsequent filtering of 149,177 heterogeneous probes based on an *I*^2^ threshold of 15% as previously suggested [70]. *P* values were adjusted for multiple comparisons using the Benjamini & Hochberg (B-H) method [71].

#### Gene annotation

Classical 450K annotations (TSS200, TSS1500, 1stExon, 5′UTR, Gene body, 3′UTR, CGI, Shelf, Shore and Open Sea) were derived from the “IlluminaHumanMethylation450kanno.ilmn12.hg19” version 0.2.1 and ChAMP version 1.8.0 Bioconductor packages. CpG islands (CGIs) were defined as GC content > 50%, observed/expected CpG ratio > 60%, and > 200 bp while CGI shore and shelves represent within and outside a 2-kb flanking region surrounding a CGI, respectively. Fisher’s exact test integrated in R (version 3.4.3) was used to estimate enrichment (alternative = “greater”) or depletion (alternative = “less”) of features of interest.

### Locus-specific validation

“True” DNA methylation analysis of a CCGG motive located at chr1: 228503770 (included in *OBSCN* locus DMR chr1: 228503693–228503882) was carried out using methylation- and glucosylation-sensitive digestions of genomic DNA. Briefly, 100 ng of genomic DNA was mixed with UDP-glucose and 4 units of 5hmC-glucosytransferase (Quest 5-hmC Detection kit, ZymoResearch), allowing 5hmC to be glucosylated (glycosyl-5hmC). Mock glucosylation consists of all of the above with the exception of glucosyltransferase. Mock- and glucosylated-DNA were subsequently incubated with either *HpaII*, *MspI* (EpiJET, ThermoFisher Scientific) or in absence of enzyme (undigested). Unmethylated genomic DNA (EpiTect Control DNA, Qiagen) was used as a negative control. Methyl-sensitive (MSRE) *HpaII* enzyme cuts only unmodified CCGG motif whereas glucosyl-sensitive (GSRE) *MspI* enzyme cleaves all modified CCGG except glucosyl-5hmC. Detection was performed by qPCR on a BioRad CFX384 Real-Time Detection System with SYBR green fluorophore and the following primers: OBSCN_F: GCTGCTGCTCAAAAACTTGC and OBSCN_R: AATGCGGACGTCACCATATC. The percentage of total 5mC + 5hmC was quantified using the 2^−ΔCt^ method comparing Mock- and *HpaII*-digested DNA with Mock- and undigested-DNA (with *MspI* as a positive control). Percentage of 5hmC was quantified by applying 2^−ΔCt^ to Glucosylated- and *MspI*-digested DNA compared to Glucosylated- and undigested-DNA. True 5mC was expressed as % (5mC + 5hmC) − % 5hmC.

### MiRNA target genes prediction

Differentially methylated loci mapping to miRNAs (UCSC Refseq annotations) from cohort 2 (including 5mC-, 5hmC- and BS-DMPs and − DMRs) were used as input for target prediction using mirDIP (version 4.1.8.2). Of note, corresponding mature miRNAs include: hsa-miR-339-5p, hsa-miR-1182, hsa-miR-1228-3p, hsa-miR-136-5p, hsa-miR-154-5p, hsa-miR-1909-3p, hsa-miR-202-3p, hsa-miR-377-3p, hsa-miR-431-5p, hsa-miR-432-5p, hsa-miR-496, hsa-miR-518, hsa-miR-548, hsa-miR-661, hsa-miR-1226-3p, hsa-miR-300, hsa-miR-802, hsa-miR-1251-5p, hsa-miR-19b-3p, hsa-miR-17-5p, hsa-miR-18a-5p, hsa-miR-19a-3p, hsa-miR-20a-5p, and hsa-miR-92a-3p. Two miRNA loci (MIR518A2 and MIR548) could not be found in the database. mirDIP integrates predictions from 30 independent resources and offers an integrative score which has been shown to provide more accuracy compared to confidence scores from the individual resources [72]. Among the predicted target genes, only the ones showing integrative scores > 0.7 were further examined using gene ontology analysis.

### Gene ontology analyses

Genomic locations of 450K probes were obtained from the ChAMP Bioconductor package version 1.8.0 [55]. Gene ontology (GO) analysis was performed using ingenuity pathway analysis (IPA) (Qiagen), applying unbiased parameters for all criteria including tissues selection. The data were analysed focusing on canonical pathways. Right-tailed Fisher’s exact test was used to calculate *P* values. Enriched GO terms with adjusted *P* values < 0.05 (Benjamini-Hochberg, B-H) were considered statistically significant. Of note, analyses of BS-generated DMPs from cohorts 1 and 2 did not result in significant pathways after B-H adjustment, and, in this case, enriched pathways with *P* values < 0.05 were shown in Additional file 4 and mentioned in the text as “modest enrichment”. We further validated findings from IPA analyses from cohort 2 using overrepresentation analysis from the online software tool WebGestalt (www.webgestalt.org) [73] under default settings and summarized using REVIGO tool [74] based on multidimensional scaling of overrepresented GO terms with semantic similarities. STRING network was generated using STRING database version 10.5.

### Brain sectioning and immunofluorescence

Brain blocks from 15 MS cases and controls (8 of them overlapping with DNA methylation analysis) were sectioned using a cryostat (Leica CM1850) at the Neurology clinic (Karolinska Hospital, Stockholm). The 14-μm-thick slices on SuperFrost slides were kept at − 20 degrees until further use. Immunofluorescence (IF) technique was utilized for examination of the transcription factor CREB in frozen MS and non-neurological control brain tissue. Rabbit monoclonal [E113] antibody specific for CREB phosphorylated on Serine 133 (1:200, Abcam #ab32096) was co-targeted with mouse monoclonal anti-NeuN antibody (Millipore #MAB377) in the samples with comparable (posterior) brain localization containing adjacent white and grey matter portions. Targets were visualized using fluorescently-labelled secondary antibodies Alexa Fluor 488 (Abcam, #ab150106) and 555 (Jackson Immunoresearch #111-545-003), respectively. NeuN^+^ white and grey matter neurons were examined for nuclear expression of phosphorylated CREB in high magnification using Zeiss LSM 700 confocal laser microscope. Captured group-representative IF- images are shown in the Fig. 5. Amount of NeuN^+^/CREB (phospho S133)^+^ WM neurons is presented as a percentage of the total amount of all detectable NeuN+ cells in the WM.

### Statistical analysis

Details of genome-wide analysis of methylation data are provided in the sections above. All correlation analysis were performed using the Spearman’s rank test. Fisher’s and Chi-square test were used for enrichment and depletion analyses. Data with more than two groups were analysed using Kruskal-Wallis test and Dunn’s test for multiple comparisons.

## Additional files

Additional file 1: **Figure S1.** Importance of separating neuronal fraction from brain samples. **Figure S2.** Neuronal subtype deconvolution. **Figure S3.** Signal distribution of BS and oxBS probes. **Figure S4.** Distribution of 5hmC *β*-values. **Figure S5.** Computational strategy of 5mC and 5hmC analyses. **Figure S6.** Comparison of 5hmC *β*-values between pipelines. **Figure S7.**Validation of *OBSCN* locus using methyl-sensitive (MSRE) and glucosyl-sensitive (GSRE) restriction enzymes. **Figure S8.** Correlation between BS and oxBS changes. **Figure S9.** Distribution of 5mC and 5hmC changes across features. **Figure S10.** Meta-analysis. **Figure S11.** Neuroanatomical localization of the samples. (PDF 2883 kb)

Additional file 2: **Table S1.** Description of cohorts. (XLSX 102 kb)

Additional file 3: **Table S2.** BS-DMPs (cohort 1, P-value <0.001). (XLSX 197 kb)

Additional file 4: **Table S3.** Canonical pathways and upstream regulators from gene ontology analysis for cohort 1, cohort 2 and meta-analysis. (XLSX 244 kb)

Additional file 5: **Table S4.** 5mC-DMPs, 5hmC-DhMPs and BS-DMPs (adjusted P-Value <0.05, cohort 2). (XLSX 1370 kb)

Additional file 6: **Table S5.** 5mC-DMRs, 5hmC-DhMRs and BS-DMRs (cohort 2). (XLSX 508 kb)

Additional file 7: **Table S6.** DMPs from meta-analysis of cohorts 1 and 2 (adjusted P-Value <0.05). (XLSX 1560 kb)

Additional file 8: **Table S7.** Comparison of DNA methylation with gene expression (Huynh *et al*., 2014). (XLSX 24 kb)

## Abbreviations

450K: Illumina Infinium Human Methylation 450K BeadChip
5hmC: DNA hydroxymethylation
5mC: DNA methylation
BS: Bisulfite
CNS: Central nervous system
CREB: cAMP response element binding
DhMP: Differentially hydroxymethylated position
DhMR: Differentially hydroxymethylated region
DMP: Differentially methylated position
DMR: Differentially methylated region
DNMT: DNA methyltransferase
GABA: Gamma-aminobutyric acid
GLU: Glutamate
GM: Grey matter
IF: Immunofluorescence
MS: Multiple sclerosis
NAWM: Normal-appearing white matter
oxBS: Oxidative bisulfite
TET: Ten-eleven translocation
TSS: Transcription starting site
UTR: Untranslated region
WM: White matter
