# Supplementary material for: Neuronal methylome reveals CREB-associated neuro-axonal impairment in multiple sclerosis
Source: Clin Epigenetics. 2019 May 30;11:86. doi: 10.1186/s13148-019-0678-1 (PMC6543588; doi:10.1186/s13148-019-0678-1)
Supplement: Supplementary file 1 — Figure S1. Importance of separating neuronal fraction from brain samples. Figure S2. Neuronal subtype deconvolution. Figure S3. Signal distribution of BS and oxBS probes. Figure S4. Distribution of 5hmC β-values. Figure S5. Computational strategy of 5mC and 5hmC analyses. Figure S6. Comparison of 5hmC β-values between pipelines. Figure S7.Validation of OBSCN locus using methyl-sensitive (MSRE) and glucosyl-sensitive (GSRE) restriction enzymes. Figure S8. Correlation between BS and oxBS changes. Figure S9. Distribution of 5mC and 5hmC changes across features. Figure S10. Meta-analysis. Figure S11. Neuroanatomical localization of the samples. (PDF 2883 kb) [file 13148_2019_678_MOESM1_ESM.pdf]

Additional file 1: Figures S1-S11

Neuronal methylome reveals CREB-associated neuro-axonal impairment in Multiple Sclerosis

Lara Kular[1], Maria Needhamsen[1], Milena Z. Adzemovic[1], Tatiana Kramarova[1], David Gomez-Cabrero[2,3,4], Ewoud Ewing[1], Eliane Piket[1], Jesper Tegnér[2,5], Stephan Beck[6], Fredrik Piehl[1,7], Lou Brundin[1,7] and Maja Jagodic[1]*.

Figure S1. Importance of separating neuronal fraction from brain samples.

Figure S2. Neuronal subtype deconvolution.

Figure S3. Signal distribution of BS and oxBS probes.

Figure S4. Distribution of 5hmC β-values.

Figure S5. Computational strategy of 5mC and 5hmC analyses.

Figure S6. Comparison of 5hmC β-values between pipelines.

Figure S7. Validation of *OBSCN* locus using methyl-sensitive (MSRE) and glucosyl-sensitive (GSRE) restriction enzymes.

Figure S8. Correlation between BS and oxBS changes.

Figure S9. Distribution of 5mC and 5hmC changes across features.

Figure S10. Meta-analysis.

Figure S11. Neuroananatomical localization of the samples.

Figure S1

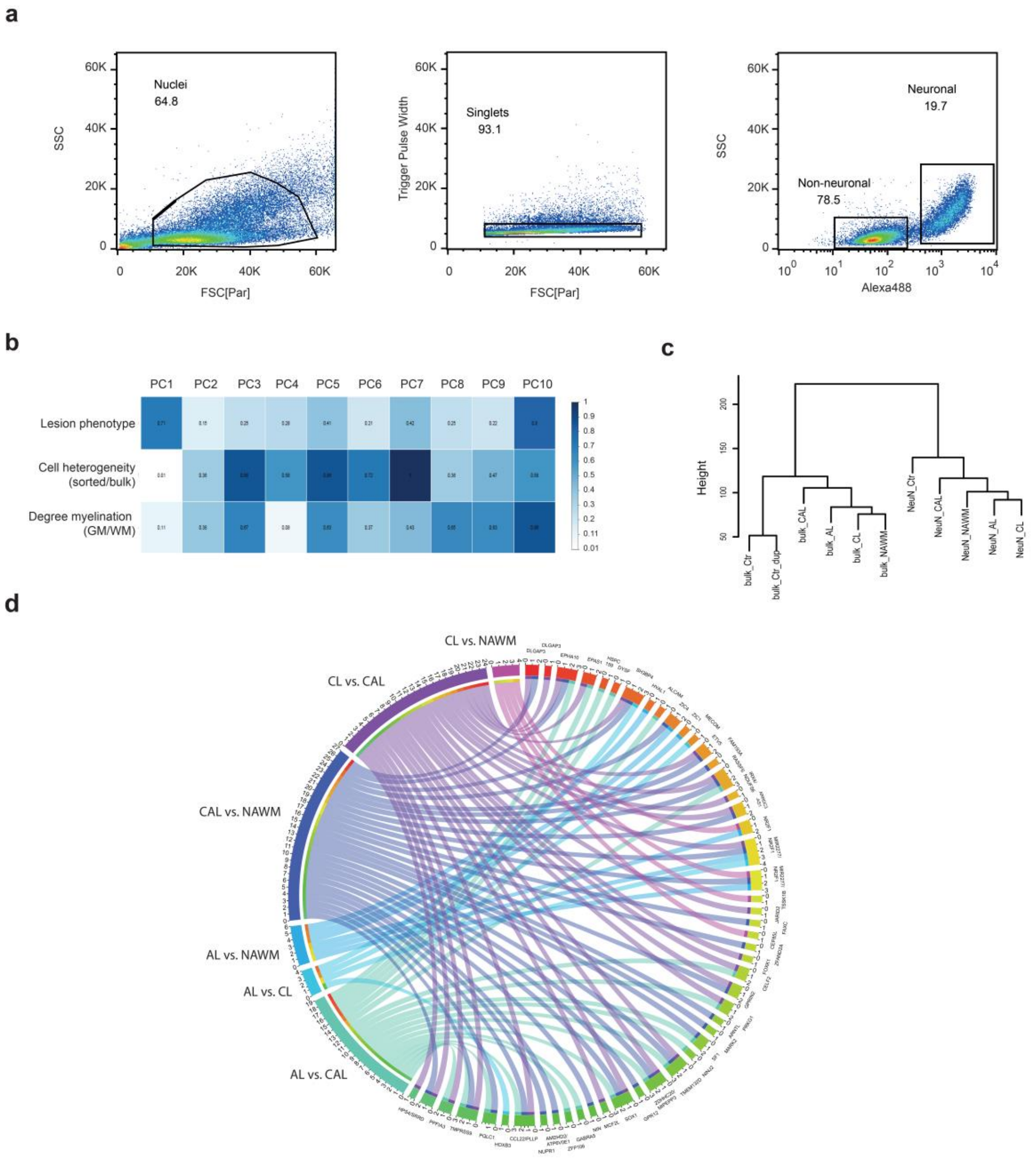

Fig. S1. Importance of separating neuronal fraction from brain samples. (**a**) Representative experiment (MS050-A2D3) illustrating the sorting strategy for separation of neuronal nuclei from brain samples by FACS: exclusion of nuclear fragment and doublets of nuclei (left, middle panels) and FACS separation of neuronal nuclei based on NeuN-staining intensity (right panel). (**b**) P-values derived from associating traits (lesion phenotype, cell heterogeneity and myelination degree) with Principal Components (PC1-PC10) of DNA methylation profiles from bulk and neuronal nuclei (NeuN) samples using Kruskal-Wallis test. (**c**) Hierarchical clustering analysis of bulk and neuronal nuclei (NeuN) from different lesion phenotypes with biological duplicate (_dup). (**d**) Differentially methylated positions (DMPs) harbouring genes associated with differences between MS lesion phenotypes after ComBat adjustment for cell heterogeneity. Each ribbon represents an association between DMP (randomly ordered and coloured red to green) and difference between lesion phenotype (ribbon colour blue to purple). The inner track recapitulates the type of association for either the DMPs or the comparison. Graduation represents the number of associations. Gene name and the type of comparison appear in the outer layer. GM, grey matter, WM, white matter, NAWM, normal appearing white matter, CL, chronic lesion, CAL, chronic active lesion, AL, active lesion, vs, versus.

Figure S2

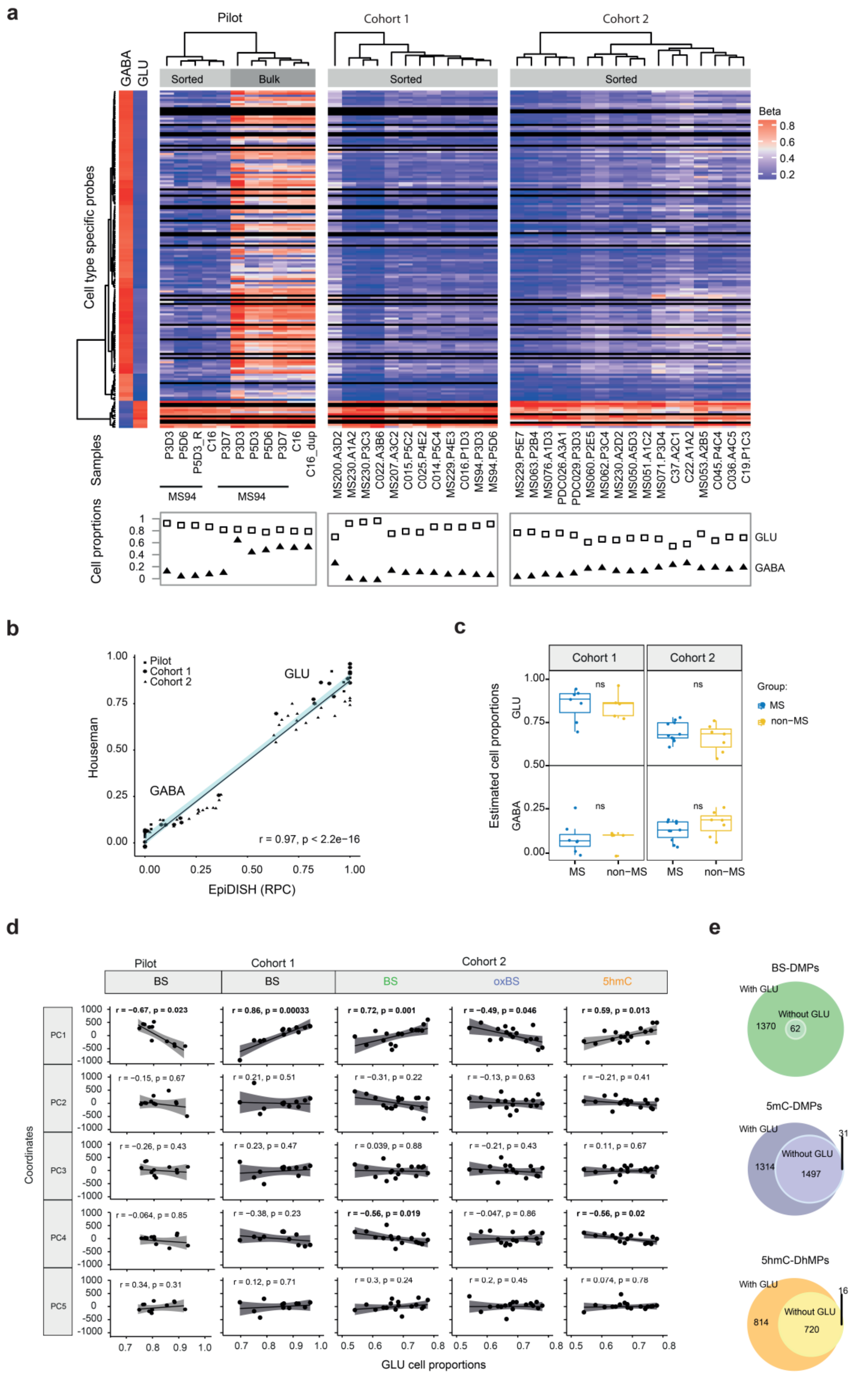
a
Pilot
Cohort 1
Cohort 2
GABA
GLU
Sorted
Bulk
Sorted
Sorted
Beta
0.8
0.6
0.4
0.2
Cell type specific probes
Samples
P3D3
P5D6
P5D3_R
C16
P3D7
P3D3
P5D3
P5D6
P3D7
C16
C16_dup
MS200.A3D2
MS230.A1A2
MS230.P3C3
C022.A3B6
MS207.A3C2
C015.P5C2
C025.P4E2
C014.P5C4
MS229.P4E3
C016.P1D3
MS94.P3D3
MS94.P5D6
MS229.P5E7
MS063.P2B4
MS076.A1D3
PDC026.A3A1
PDC029.P3D3
MS060.P2E5
MS062.P3C4
MS230.A2D2
MS050.A5D3
MS051.A1C2
MS071.P3D4
C37.A2C1
C22.A1A2
MS053.A2B5
C045.P4C4
C036.A4C5
C19.P1C3
MS94
MS94
Cell proprtions
GLU
GABA
b
Pilot
Cohort 1
Cohort 2
GLU
GABA
Houseman
EpiDISH (RPC)
r = 0.97, p < 2.2e−16
c
Cohort 1
Cohort 2
Estimated cell proportions
GLU
GABA
ns
Group:
MS
non−MS
d
Pilot
Cohort 1
Cohort 2
BS
BS
BS
oxBS
5hmC
PC1
PC2
PC3
PC4
PC5
Coordinates
GLU cell proportions
r = −0.67, p = 0.023
r = 0.86, p = 0.00033
r = 0.72, p = 0.001
r = −0.49, p = 0.046
r = 0.59, p = 0.013
r = −0.15, p = 0.67
r = 0.21, p = 0.51
r = −0.31, p = 0.22
r = −0.13, p = 0.63
r = −0.21, p = 0.41
r = −0.26, p = 0.43
r = 0.23, p = 0.47
r = 0.039, p = 0.88
r = −0.21, p = 0.43
r = 0.11, p = 0.67
r = −0.064, p = 0.85
r = −0.38, p = 0.23
r = −0.56, p = 0.019
r = −0.047, p = 0.86
r = −0.56, p = 0.02
r = 0.34, p = 0.31
r = 0.12, p = 0.71
r = 0.3, p = 0.24
r = 0.2, p = 0.45
r = 0.074, p = 0.78
e
BS-DMPs
With GLU
Without GLU
1370
62
5mC-DMPs
With GLU
Without GLU
31
1314
1497
5hmC-DhMPs
With GLU
Without GLU
16
814
720

Fig. S2. Neuronal subtype deconvolution. (**a**) Heat maps visualizing DNA methylation levels (β) of 162 cell type specific CpG sites used in the reference-based Houseman algorithm for estimation of GABA (black triangles) and GLU (white squares) cell proportions. Pilot samples, cohorts 1 and 2 all contained sorted cells (light grey), whereas the pilot samples further included bulk cells (dark grey). Sample name and brain location are given for each sample. (**b**) Scatter plot of GABA and GLU cell proportions estimated by EpiDISH RPC and Houseman's reference-based methods for the pilot samples (squares), cohort 1 (asterisk) and cohort 2 (triangle). Spearman's rank correlation coefficient (r) and P-values (p) are given. (**c**) Boxplots of estimated GLU and GABA cell proportions in cohorts 1 and 2 between cases (blue) and controls (yellow). P-values from non-paired t-tests are given. (**d**) Scatterplots of GLU cell proportions and pilot samples, cohorts 1 and 2 (BS, oxBS or 5mC, and 5hmC) principal component (PCs) coordinates derived from Swan and Combat-corrected beta-values, respectively. Regression line, confidence interval, P-value (p) and Spearman's rank correlation coefficient (r) are given. Significant correlations ($p < 0.05$) are highlighted in bold. (**e**) Venn Diagrams of BS-DMPs, 5mC-DMPs and 5hmC-DhMPs (adj.P.val < 0.05) from cohort 2 identified with or without including GLU proportions in the statistical model.

Figure S3

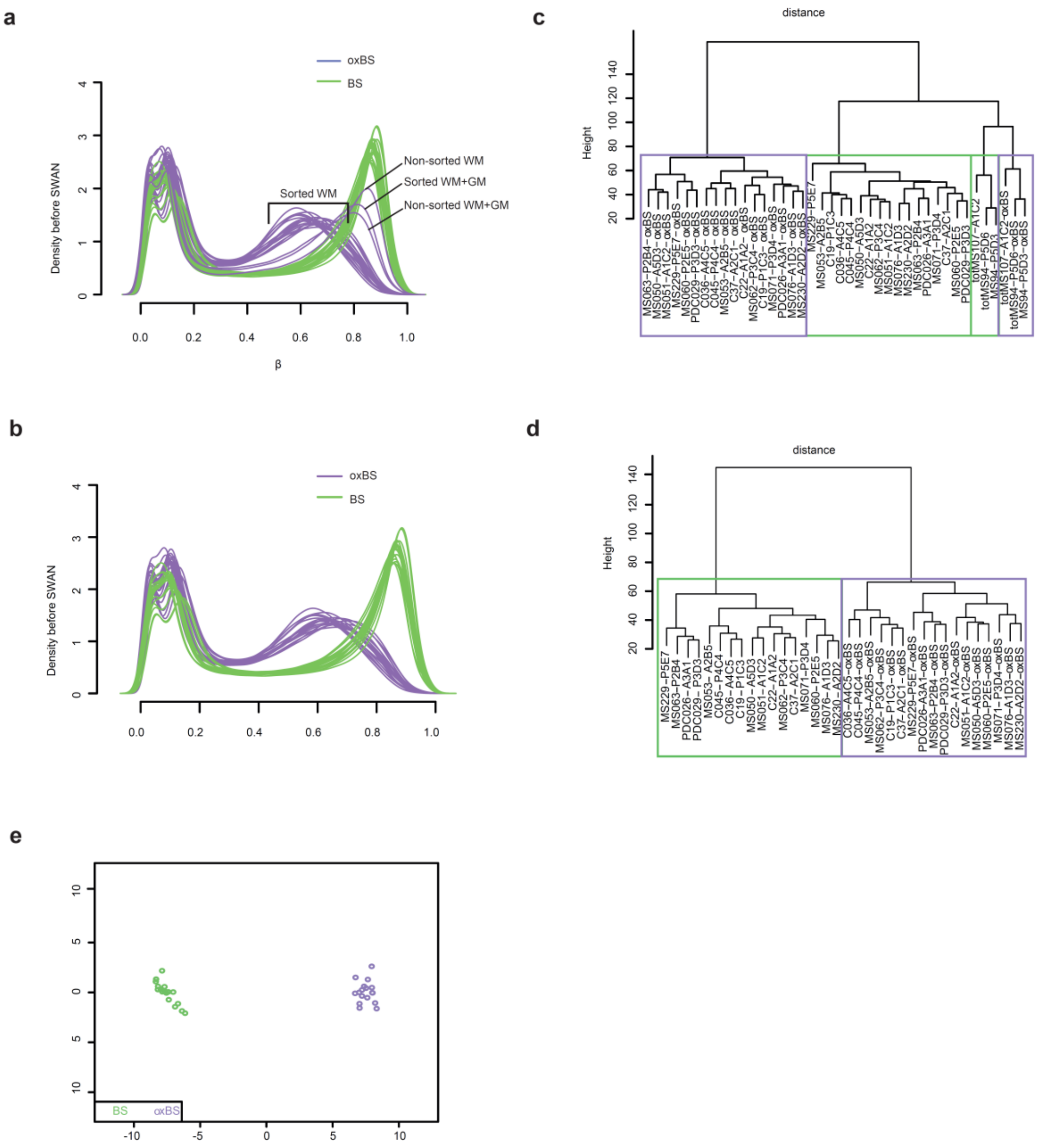

Fig. S3. Signal distribution of BS and oxBS probes. Density plots of oxBS (purple) and BS (green) β-values before SWAN-based type I/ll normalization, including (**a**) and excluding (**b**) non-sorted bulk samples as well as mixed white matter (WM) and grey matter (GM) tissue. Hierarchical clustering analysis of (**c**) NeuN$^+$-neuronal nuclei sorted from WM as well as non-sorted bulk samples and mixed white matter (WM) and grey matter (GM) tissue and (**d**) of NeuN$^+$-neuronal nuclei sorted from WM only. (**e**) Multidimensional scaling (MDS) of the 1000 most variable SWAN-normalized oxBS and BS positions. BS and oxBS signals are depicted in green and purple, respectively.

Figure S4

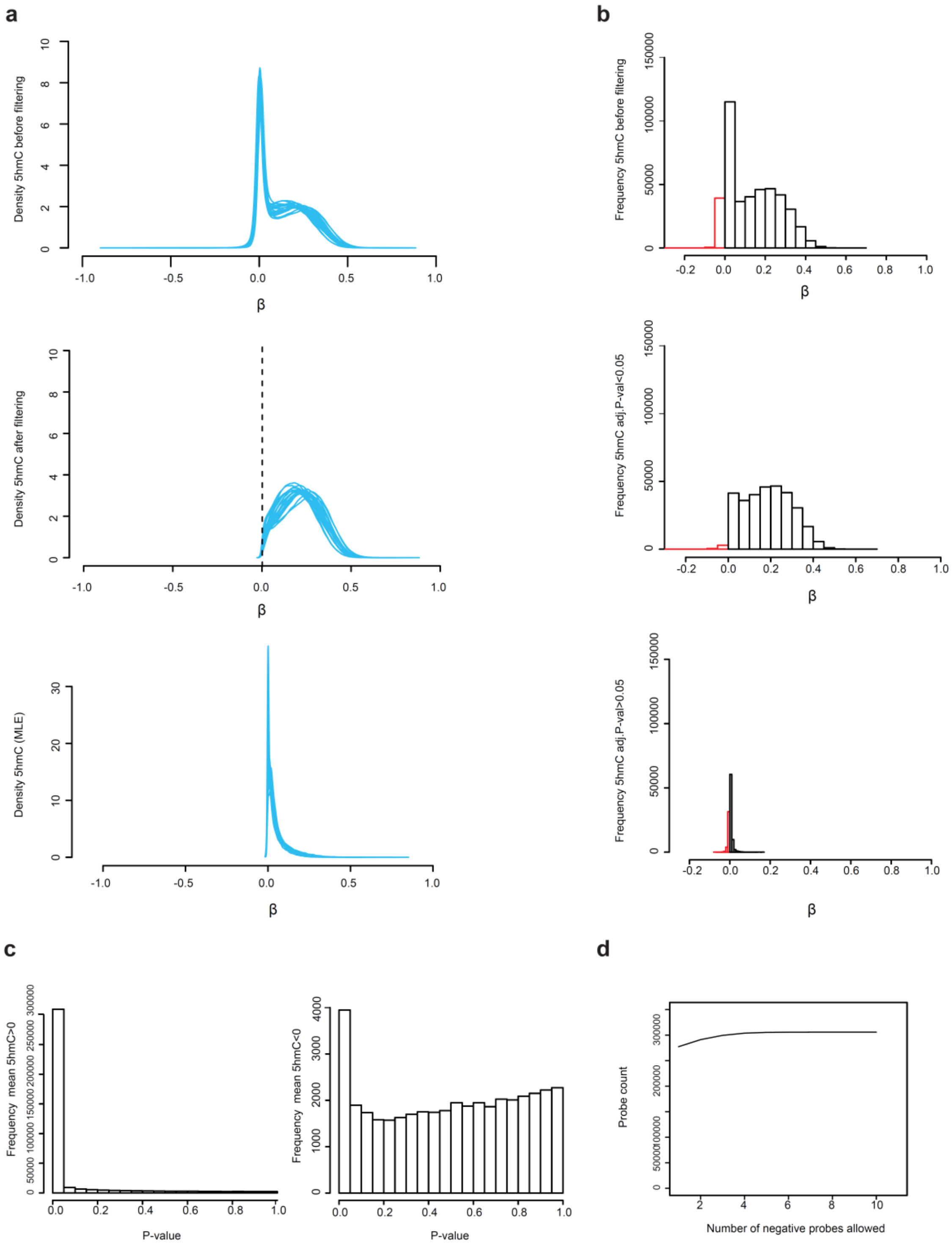

Fig. S4. Distribution of 5hmC β-values. (**a**) Density plots of 5hmC β-values (β_{BS}-β_{oxBS}) before probe filter (upper panel), after probe filter (middle panel). Lower panel: Reintroduced probes processed through the oxyBS pipeline30 relying on maximum likelihood estimation (MLE). (**b**) Distribution of all (β_{BS}-β_{oxBS}) 5hmC β-values (upper panel), significant (adj.P.Val < 0.05) 5hmC β-values (middle panel) and non-significant (adj.P.Val > 0.05) 5hmC β-values (lower panel). Red bars indicate negative 5hmC β-values. Significant (adj.P.Val < 0.05) 5hmC β-values were estimated using champ.TrueMethyl function (BS vs. oxBS). (**c**) Distribution of P-values of 5hmC sites with positive (left panel) and negative (right panel) mean 5hmC β-values. (**d**) Probe count depending on the number of negative β-values allowed.

Figure S5

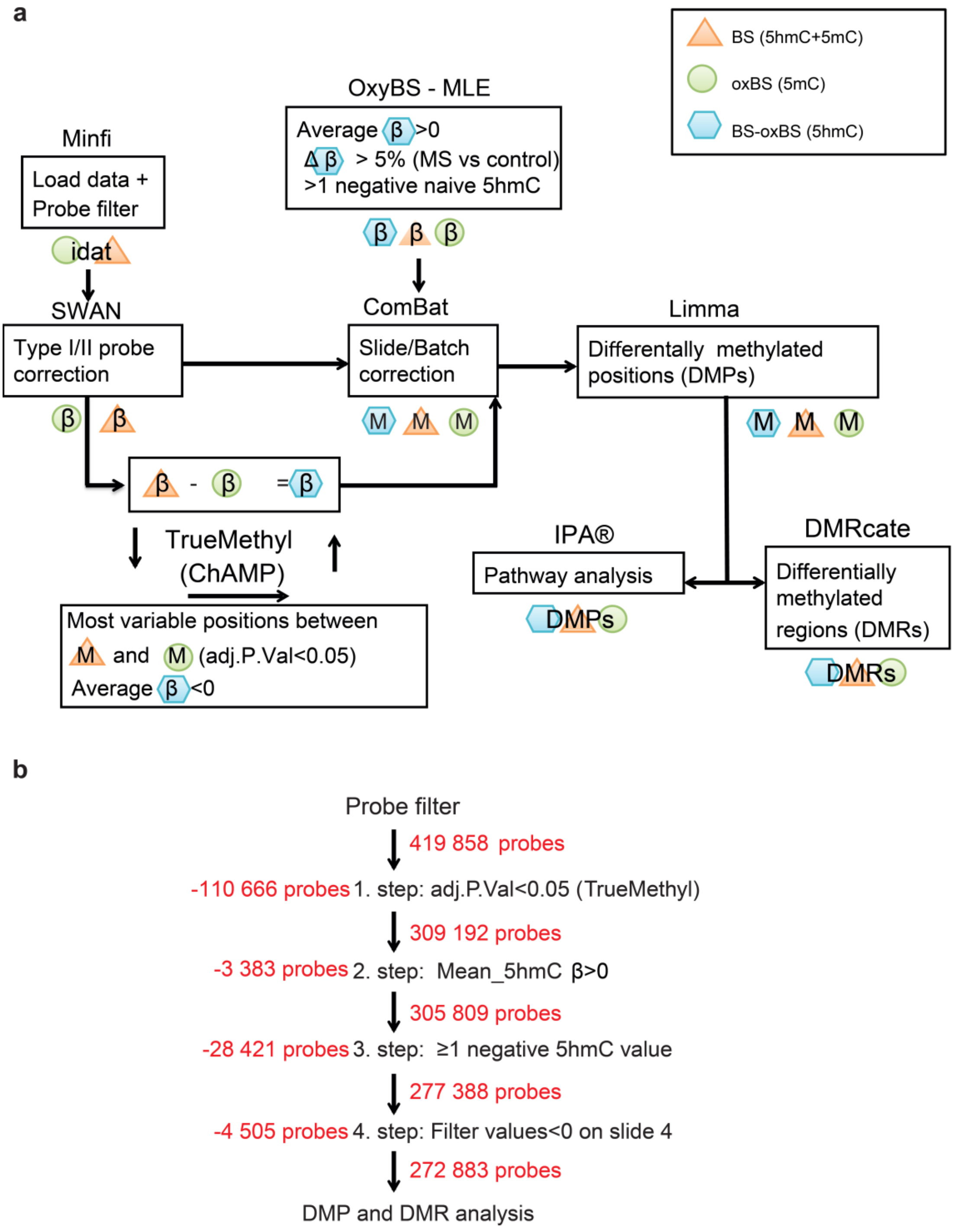

Fig. S5. Computational strategy of 5mC and 5hmC analyses. (**a**) Overview of 5hmC analysis pipeline. (**b**) Workflow of 5hmC probes stringent filter strategy.

Figure S6

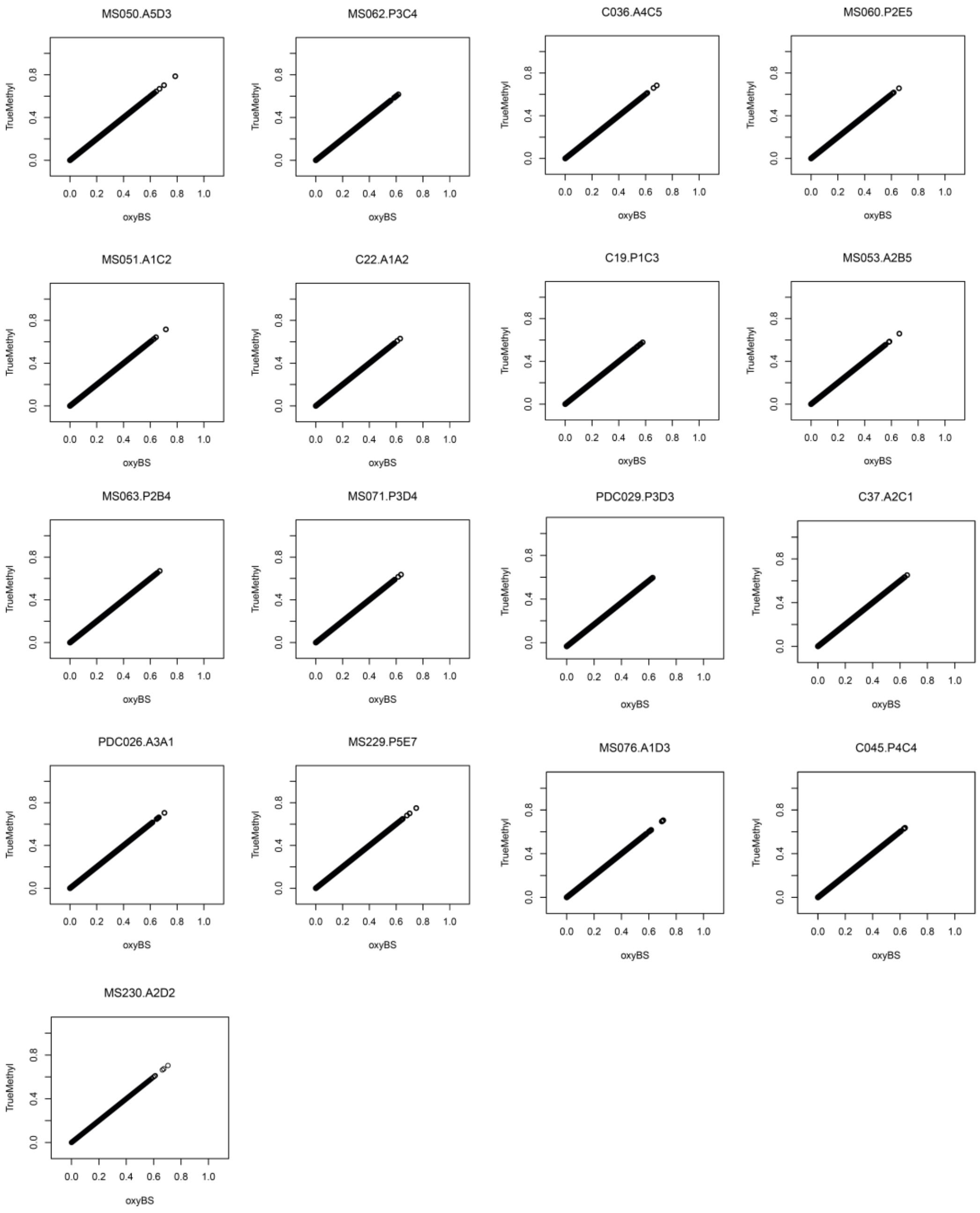

Fig. S6. Comparison of 5hmC β-values between pipelines. Scatter plots comparing (β_{BS} - β_{oxBS}) 5hmC β-values with Maximum Likelihood Estimated 5hmC β-values from the oxyBS pipeline for each sample.

Figure S7

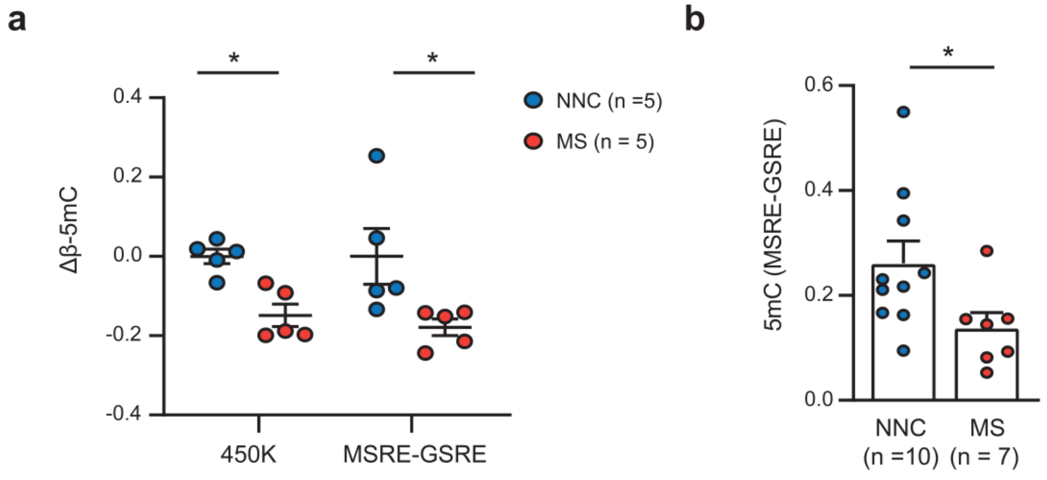

Fig. S7. Validation of *OBSCN* locus using methyl-sensitive (MSRE) and glucosyl-sensitive (GSRE) restriction enzymes. a. True methylation changes (Δβ-5mC) obtained using oxBS-450K (cg18857768) or with MSRE-GSRE (chr1:228503770), included in *OBSCN* gene DMR (chr1:228503693-228503882), in 5 non-neurological controls (NNC, blue circles) and 5 Multiple Sclerosis patients (MS, red circles) from cohort 2. **b.** Percentage of 5mC in an extended cohort of 10 NNC and 7 MS, including the aforementioned 10 samples overlapping with cohort 2. * $p < 0.05$ using unpaired *t*-test.

Figure S8

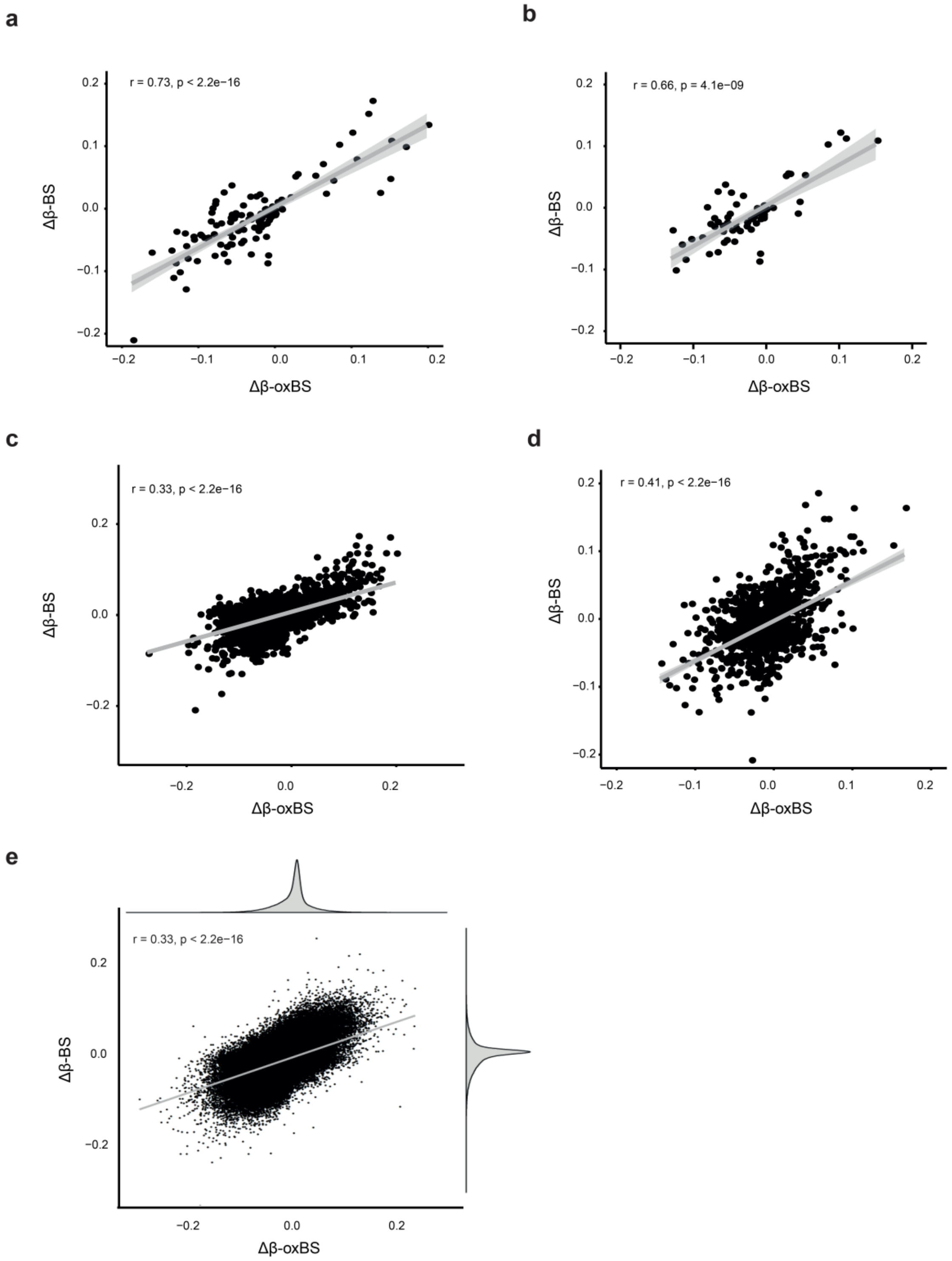

Fig. S8. Correlation between BS and oxBS changes. Scattered plots of oxBS-Δβ and BS-Δβ at overlapping sites: 119 sites at BS P-Value < 0.001 and oxBS-adjusted P-Value < 0.05 (**a**) and 64 sites at BS-adjusted P-Value < 0.001 and oxBS-adjusted P-Value < 0.05 (**b**). Scattered plots of oxBS-Δβ and BS-Δβ at all significant oxBS-DMPs (adjusted P-Value < 0.05) (**c**), at all significant BS-DMPs (adjusted P-Value < 0.05) (**d**) and at all sites (**e**). Spearman's rank correlation coefficient (r) and P-values (p) are given.

Figure S9

a

	5mC-DMPs (P-value)		5hmC-DhMPs (P-value)	
	enrichment	depletion	enrichment	depletion
**Gene features**				
TSS1500	1.00E+00	**4.24E-23**	9.87E-01	1.69E-02
TSS200	1.00E+00	**9.38E-12**	3.83E-04	1.00E+00
5UTR	1.00E+00	4.31E-05	9.69E-01	4.13E-02
1stExon	9.46E-01	7.25E-02	3.86E-02	9.76E-01
Body	**1.16E-39**	1.00E+00	1.44E-01	8.71E-01
3UTR	7.45E-04	9.99E-01	9.65E-01	4.76E-02
**CGI features**				
Island	1.00E+00	**3.27E-09**	**1.85E-07**	1.00E+00
Shore	9.98E-01	2.03E-03	8.97E-01	1.14E-01
Shelf	3.04E-04	1.00E+00	9.97E-01	3.91E-03
Open sea	**1.10E-09**	1.00E+00	8.76E-01	1.35E-01

b

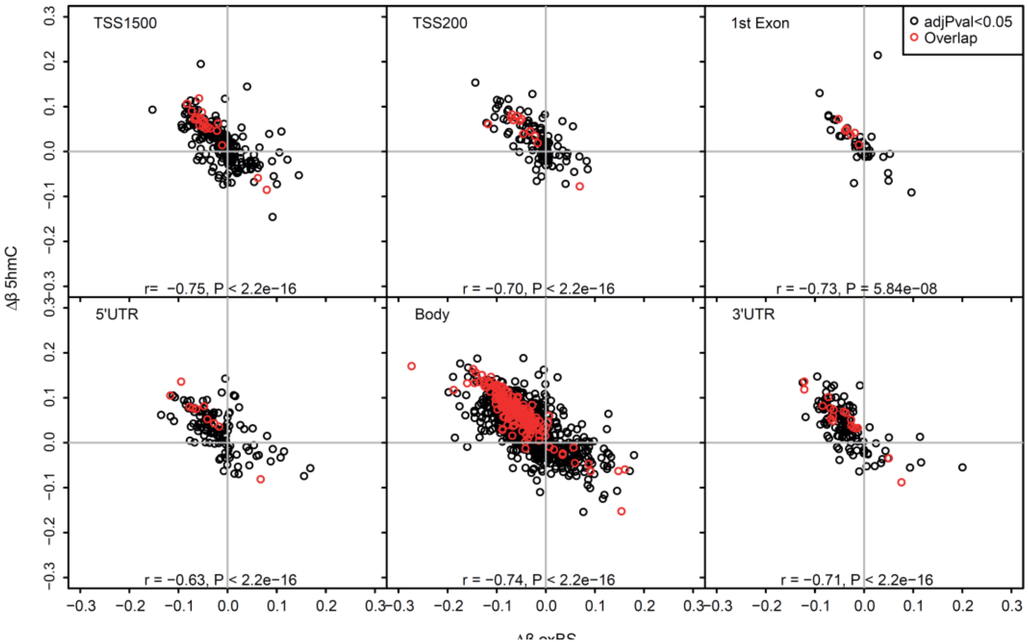

Fig. S9. Distribution of 5mC and 5hmC changes across features. (**a**) Enrichment and depletion analyses of DMPs and DhMPs (adjusted P-value < 0.05) for gene- and CpG Island (CGI)-related features. P-values from Fisher's exact tests < 1.00E-05 are highlighted in bold. (**b**) Scatter plots comparing 5mC-Δβ (oxBS) and (βBS - βoxBS) 5hmC-Δβ of sites overlapping with classical HM450K gene features. Black circles represent all significant sites (adjusted P-value < 0.05, MS vs. non-neurological controls), for either 5mC or 5hmC, whereas red circles represent overlapping sites with adj.P-value < 0.05 (MS vs. non-neurological controls) for both 5mC and 5hmC.

Figure S10

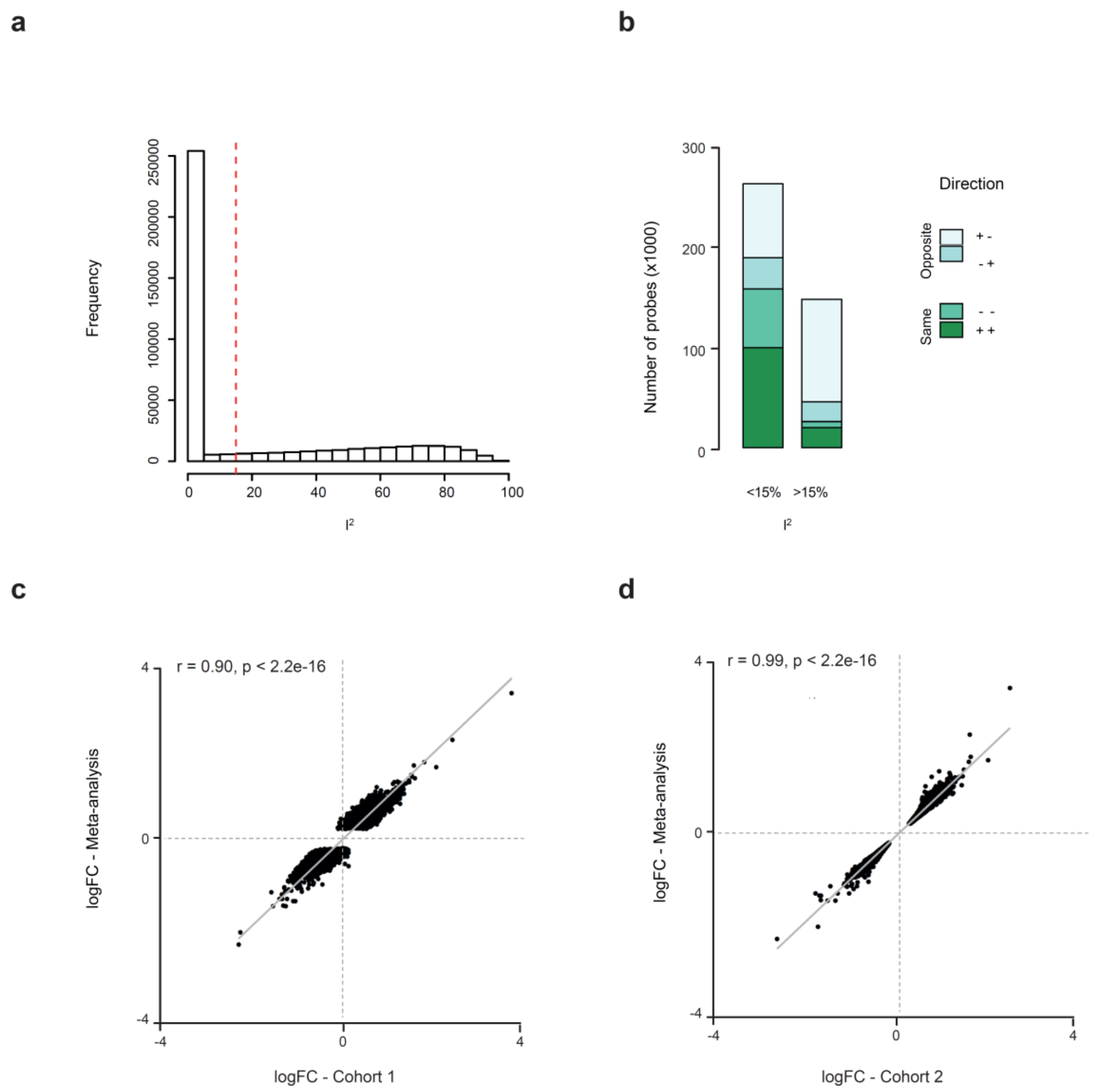

Fig. S10. Meta-analysis. (**a**) Distribution of probes depending on the I^2 estimate of heterogeneity. Out 414,306 common cohorts 1 and 2 BS-derived probes, 265,129 were considered homogenous between the two studies (based on a I^2-threshold of 15%). (**b**) Distribution of homogenous (I^2 < 15%) and heterogeneous (I^2 > 15%) probes showing opposite or same direction between cohorts 1 and 2. The I^2 filter mainly removed probes with effect sizes of opposite directions between the two cohorts. Scatter plots showing logFoldChange (logFC on M values) between probes from cohorts 1 (**c**) or cohort 2 (**d**) and BS-DMPs from meta-analysis. Regression line, Spearman's rank correlation coefficient (r) and P-values (p) are given.

Figure S11

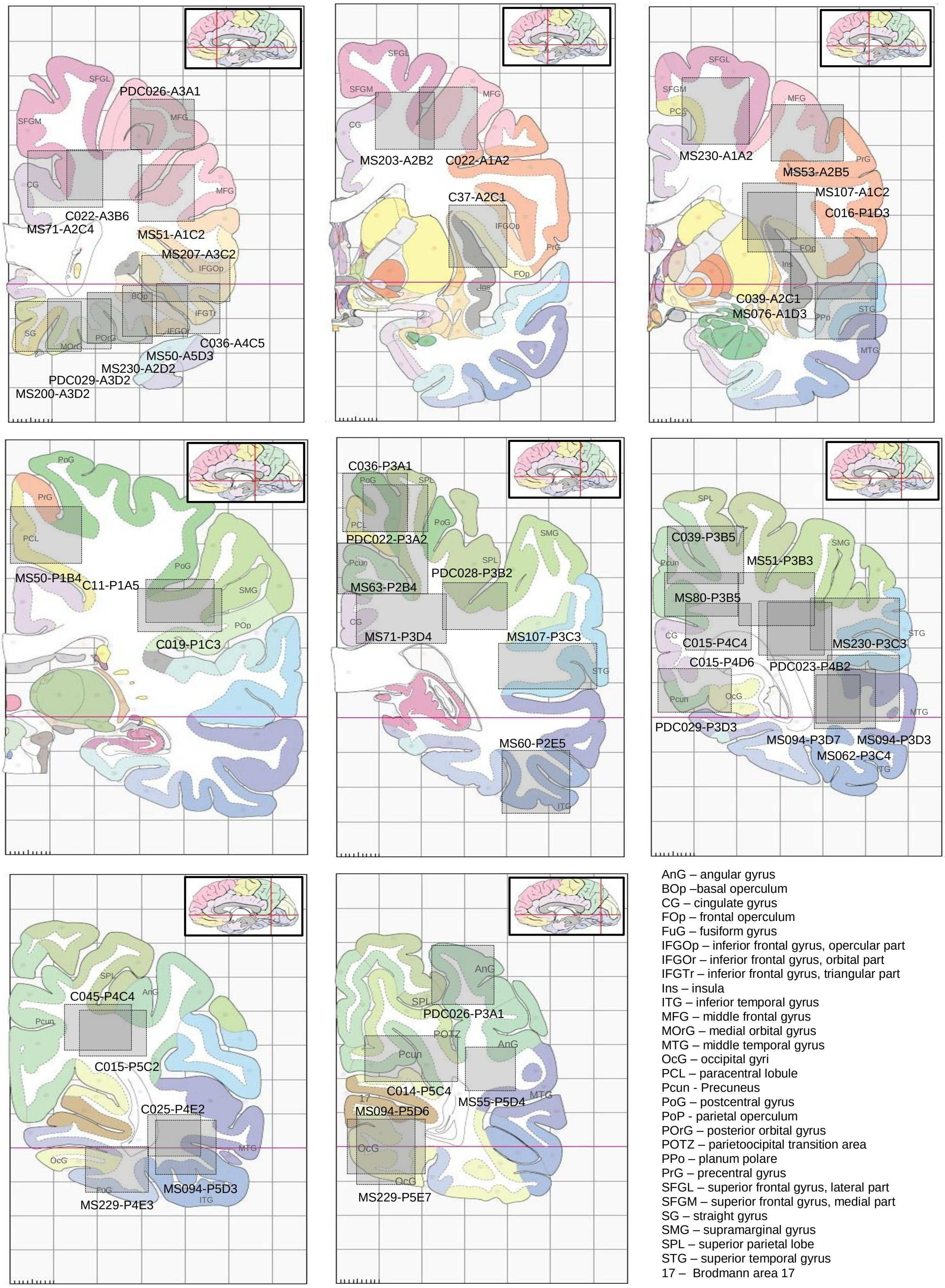

AnG – angular gyrus
BOp –basal operculum
CG – cingulate gyrus
FOp – frontal operculum
FuG – fusiform gyrus
IFGOp – inferior frontal gyrus, opercular part
IFGOr – inferior frontal gyrus, orbital part
IFGTr – inferior frontal gyrus, triangular part
Ins – insula
ITG – inferior temporal gyrus
MFG – middle frontal gyrus
MOrG – medial orbital gyrus
MTG – middle temporal gyrus
OcG – occipital gyri
PCL – paracentral lobule
Pcun - Precuneus
PoG – postcentral gyrus
PoP - parietal operculum
POrG – posterior orbital gyrus
POTZ – parietooccipital transition area
PPo – planum polare
PrG – precentral gyrus
SFGL – superior frontal gyrus, lateral part
SFGM – superior frontal gyrus, medial part
SG – straight gyrus
SMG – supramarginal gyrus
SPL – superior parietal lobe
STG – superior temporal gyrus
17 – Brodmann area 17

Figure S11. Neuroanatomical localization of the samples.
For genome-wide and locus-specific methylation analyses, neuronal nuclei were sorted from the white matter. Brain sections used for immunofluorescence include all structures. Brain blocks, with images provided by the biobank, were mapped using the human brain atlas (http://www.thehumanbrain.info/).
